# Microdiversity and fine-scale niche differentiation support persistence and coexistence of acidophiles in acid mine drainage

**DOI:** 10.3389/fmicb.2025.1697424

**Published:** 2025-12-03

**Authors:** Alejandro Palomo, Bowei Li, Zhixiong Huang, Yunjie Ma, Wenle Peng, Weishi Wang, Yan Zheng, Yi Wen, Lihong Yang

**Affiliations:** 1 State Key Laboratory of Soil Pollution Control and Safety, School of Environmental Science and Engineering, Southern University of Science and Technology, Shenzhen, China; 2 Guangdong-Hong Kong Joint Laboratory for Soil and Groundwater Pollution Control, School of Environmental Science and Engineering, Southern University of Science and Technology, Shenzhen, China; 3 Key Laboratory of Integrated Surface Water-Groundwater Pollution Control of the Ministry of Ecology and Environment, School of Environmental Science and Engineering, Southern University of Science and Technology, Shenzhen, China; 4 Technical Centre for Soil, Agriculture and Rural Ecology and Environment, Ministry of Ecology and Environment, Beijing, China

**Keywords:** acid mine drainage (AMD), acidophiles, microbial diversity, niche differentiation, community assembly, 16S rRNA gene amplicon sequencing

## Abstract

**Introduction:**

Acid mine drainage (AMD) systems are extreme acidic environments characterized by low pH and high metal concentrations that shape unique microbial ecosystems. While acidophilic microorganisms are known to drive AMD biogeochemistry, the ecological processes governing their community assembly, niche partitioning, and long-term stability remain incompletely resolved.

**Methods:**

To investigate microbial diversity, community structure, and assembly mechanisms, we integrated high-resolution 16S rRNA gene amplicon sequencing with community ecology analyses and null modeling approaches in an AMD system located in Zhejiang Province, China. We examined microbial communities across water and sediment habitats, assessing the influence of environmental variables (e.g., pH, metal concentrations, redox potential) on community composition. Null models were used to quantify the relative roles of deterministic and stochastic processes in community assembly.

**Results and discussion:**

Community structure was primarily shaped by pH and habitat type (water vs. sediment), with low-pH conditions selecting for persistent, abundant taxa dominated by specialized acidophiles. Within this group, we identified significant fine-scale niche partitioning and intra-genus microdiversity, both of which were associated with greater persistence across heterogeneous conditions. Co-occurrence and niche analyses revealed that closely related taxa often occupy distinct ecological niches structured by gradients of metals, redox potential, and oxygen availability. Ecological assembly modeling indicated that deterministic homogeneous selection and stochastic drift dominate under the harshest conditions. In contrast, dispersal limitation becomes more important in less chemically-stressed sites, indicating that spatial constraints gain importance when environmental filtering weakens. Our findings reveal that AMD microbial communities are shaped by a dynamic interplay between strong environmental selection, stochasticity, and spatial factors. These insights advance fundamental understanding of microbial community organization in extreme habitats and have practical implications for predicting ecosystem responses to environmental change and optimizing bioremediation strategies in contaminated systems.

## Introduction

Acid mine drainage (AMD) represents one of the most pervasive and persistent environmental challenges associated with mining worldwide, affecting thousands of sites across multiple continents and generating acidic, metal-rich effluents that can persist for centuries (Nordstrom and Alpers, 1999). These environments, often with low pH values and high concentrations of dissolved metals such as iron, manganese, and zinc, are typically hostile to most forms of life. Yet paradoxically, they support diverse microbial communities that not only survive but actively drive the geochemical reactions underpinning AMD formation (Baker-Austin and Dopson, 2007). Acidophilic microorganisms mediate the oxidative dissolution of sulfide minerals, accelerating acid and metal release, while also performing essential roles in sulfur and iron cycling. Over the past two decades, molecular and genomic advances have revealed that AMD systems harbor remarkable microbial diversity, including both well-characterized acidophiles (e.g., *Leptospirillum, Acidithiobacillus, Ferroplasma*) and novel, uncultured taxa with poorly understood metabolisms (Baker and Banfield, 2003; Chen et al., 2016; Grettenberger and Hamilton, 2021). These discoveries have expanded our understanding of life at environmental extremes and emphasized the central role of microorganisms in both the generation and potential remediation of AMD (Johnson and Hallberg, 2005).

Considerable progress has been made in describing microbial composition and metabolic activities in AMD systems (Luo et al., 2023; Huang et al., 2025). Nevertheless, fundamental ecological questions remain unresolved: it is unclear which processes determine species coexistence, how microbial taxa partition available niches, and what mechanisms maintain community stability across steep environmental gradients (Chang et al., 2023; Orr et al., 2025). Addressing these gaps is critical for predicting microbial responses to environmental change and informing effective bioremediation strategies (Hanson et al., 2012; Nemergut et al., 2013).

Microbial community assembly is governed by a combination of deterministic processes, including environmental filtering and competitive interactions; and stochastic processes, such as dispersal limitation and random demographic fluctuations (Dumbrell et al., 2010; Vellend, 2010; Stegen et al., 2012). In AMD systems, extreme acidity and high metal concentrations strongly filter taxa (Kuang et al., 2017; Teng et al., 2017), favoring highly specialized acidophiles, while stochastic drift may influence community composition in smaller or isolated populations (Jones et al., 2023). Despite their recognized importance, the relative contributions of these processes in AMD environments remains poorly resolved, constraining our ability to predict how these extreme microbial communities will respond to environmental fluctuations or remediation efforts (Chen et al., 2016). Furthermore, while pH has been established as a primary driver of community composition in acidic environments (Chen et al., 2013; Liu et al., 2014), the fine-scale niche differentiation that enables coexistence of closely related acidophiles remains largely unexplored, preventing a full understanding of microdiversity patterns and their ecological implications.

To address these gaps, we investigated how environmental gradients shape microbial community structure, assembly mechanisms, and niche differentiation in a spatially heterogeneous AMD system in Zhejiang province, China. We hypothesized that extreme acidity and metal concentrations would intensify deterministic assembly, whereas stochastic processes would still influence community composition in less extreme microhabitats. Using high-resolution amplicon sequencing combined with phylogeny-informed null modeling and network analyses, we quantified assembly processes, explored microdiversity patterns, and identified fine-scale habitat differentiation between water and sediment communities. This study provides novel insights into the ecological principles governing microbial life under extreme stress, clarifies the interplay between deterministic and stochastic processes in AMD, and enhances our capacity to predict microbial community responses to environmental change and guide bioremediation efforts.

## Materials and methods

### Study site

**Figure 1:**
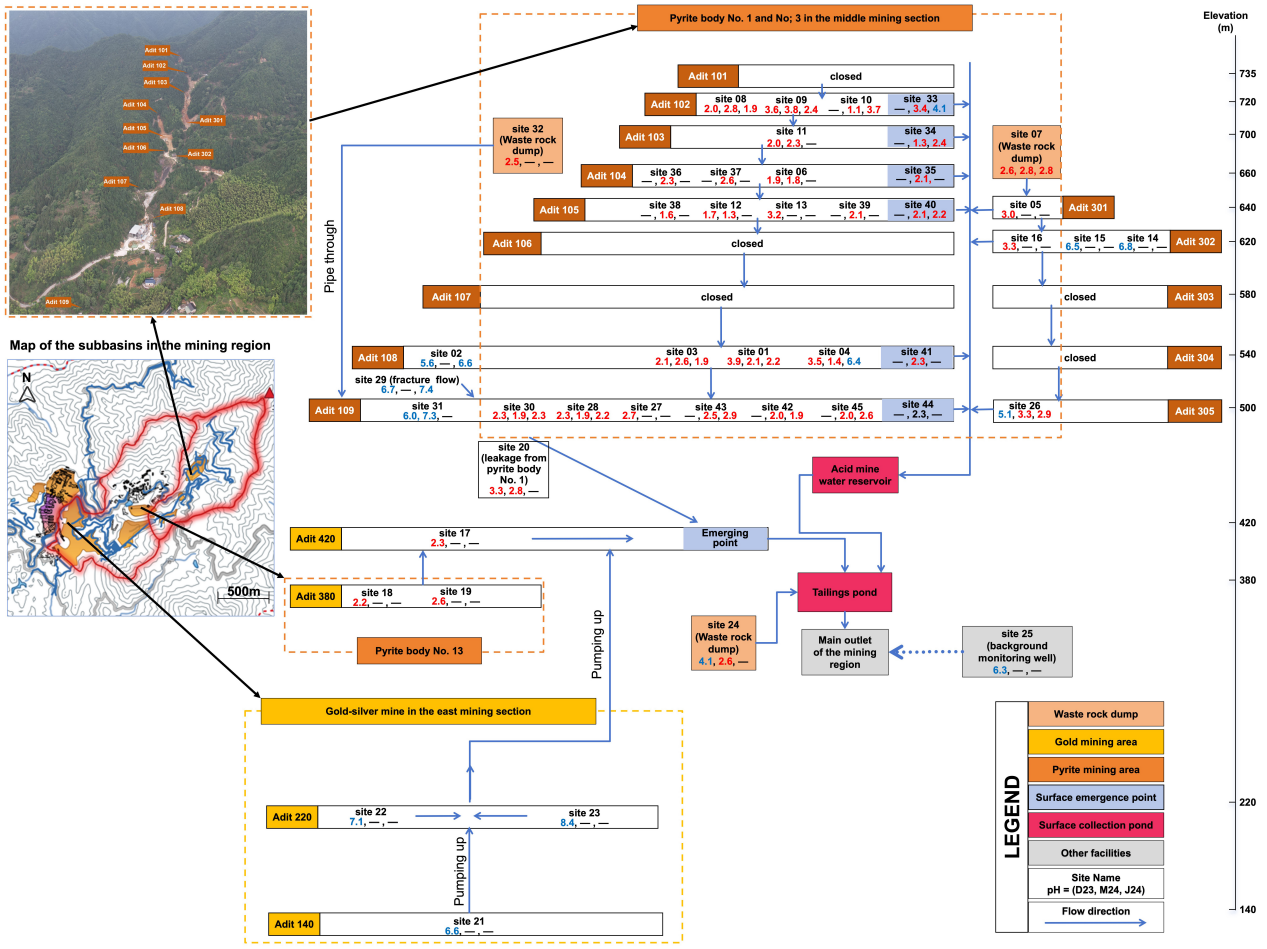
Map of the mining area showing the locations of sampling sites. For each site, the pH of the sample is shown for the three sampling times (“—” denotes samples was not collected at that time), with red color denoting low pH (<4), and blue color higher pH (>4).

The study was conducted in the Suichang mining area, located in Suichang County, Lishui City, Zhejiang Province, China. The Suichang Mine is situated in the southwestern part of the Shaoxing-Longquan mineral belt. The area contains significant pyrite, gold, and silver deposits, along with medium-sized lead-zinc ore deposits, primarily hosted in metamorphic and volcanic rocks. Mining activities in the region have a long history, dating back to approximately 1,800, with initial large-scale pyrite extraction beginning in 1954 and ceasing in 1976. Following the cessation of mining operations in the pyrite section, extensive underground voids and tunnels were abandoned. Rainwater infiltrates into waste rock dumps in the valley or through shallower fractures into voids and tunnels, leading to the dissolution and oxidation of sulfide minerals. This process generates approximately 600,000 m3 of acidic mine drainage annually, characterized by low pH and elevated concentrations of heavy metals. The mining area spans from elevation 460 m to 735 m with a network of tunnels in 10 levels. Sampling was conducted at various locations, including inside mine tunnels (from small streams, dripping points, ponds, and reservoirs), at tunnel exits (from discharging streams), and outside the tunnels (in tailings ponds, water storage areas, and surrounding groundwater) (Figure 1).

### Sample collection

Surface water (~3 L) was collected below the air-water interface, and sediments (top 2 cm) were sampled simultaneously from the same site. In total, 65 water and 47 sediment samples were collected (Figure 1, Supplementary Table S1). Water was pre-filtered through an 8 μm mixed-cellulose-ester (MCE) membrane (Merck Millipore, Ireland) to remove coarse particles, then filtered through a 0.22 μm MCE membrane (Merck Millipore, Ireland) using a peristaltic pump. Filters from each sample were transferred to sterile 50 mL tubes containing 10 mL of ice-cold sterile PBS, vortexed at maximum speed (30,000 rpm) for 10 min to detach microbial cells, and the filters were then removed with flame-sterilized forceps. The resulting eluate was centrifuged at 4,400 × g for 40 min at 4 °C, the supernatant was discarded, and the pellet was resuspended in 2 mL of fresh sterile PBS for downstream DNA extraction.

### DNA extraction, sequencing, and sequence analysis

Total genomic DNA was extracted from the processed water samples and from the sediments (0.5 g) using the CTAB/SDS approach. DNA integrity and concentration were verified by 1 % agarose-gel electrophoresis. The V4-V5 hypervariable region of the 16S rRNA gene was amplified from the extracted DNA with the 515F-Y 5′-GTGYCAGCMGCCGCGGTAA3′) and the 926R 5′-CCGYCAATTYMTTTRAGTTT3′) primers (Parada et al., 2016). Amplicon libraries were prepared with the NEBNext^®^ Ultra™ II DNA Library Prep Kit (New England Biolabs) and sequenced on an Illumina NovaSeq 6000 platform (2 × 250 bp paired-end reads) by Novogene Co., Ltd. (Beijing, China). Quality control, trimming, merging of paired ends, and error correction were performed in DADA2, which outputs the abundance of error-corrected amplicon sequence variants (Callahan et al., 2016). Comparison of amplicon sequence variants with the GTDB database r220 (Parks et al., 2022) was used for taxonomic assignment of 16S ribosomal RNA genes.

### Persistent abundant taxa and microdiversity analyses

Following the framework of Auladell et al. (2022), an ASV was designated abundant if its relative abundance reached ≥ 1% in at least one sample, while an ASV that never surpassed this threshold was considered permanently rare. In addition, ASVs were further classified into three distribution categories based on their occupancy across samples: broad-distribution (≥75% occurrence), intermediate-distribution (>10% and < 75%), and narrow-distribution ( ≤ 10%). Thus, we define persistent abundant taxa as ASVs that occur in ≥75% of samples and achieve ≥1% relative abundance in at least one sample, representing taxa that maintain consistent presence across environmental gradients while achieving ecological significance through abundance. Finally, all abundant ASVs were evaluated as Conditionally Rare Taxa (CRT), which are typically rare ASVs that occasionally become highly abundant (bimodality = 0.9, relative abundance ≥1% in at least one sample).

For microdiversity analysis, we followed the approach described by García-García et al. (2019). First, ASVs were clustered into 98.7%-similarity OTUs using VSEARCH (Rognes et al., 2016). Only OTUs whose cumulative read count across all samples exceeded 5,000 were retained, as below this threshold the Shannon index is unreliable. For each qualifying OTU, the abundances of its member ASVs were rarefied to exactly 5,000 reads to remove the influence of global abundance. Effective micro-diversity was then calculated as exp(Shannon) on the rarefied ASV vector, yielding the effective number of distinct ASVs within that OTU. To quantify ecological behavior, the same rarefied counts were used to determine persistence (fraction of samples with ≥1 count) and variability (coefficient of variation of abundance across samples).

### Niche preference analysis

To investigate whether taxa within a genus exhibit covariation, potentially indicating a shared realized spatio-temporal niche, we applied the approach of Auladell et al. (2022). Prior to analysis, we applied several data preprocessing and filtering steps to ensure robust proportionality calculations: (1) ASVs with zero counts across all samples within each genus were removed; (2) samples with zero counts for all ASVs within a genus were excluded; (3) only ASVs present in at least 5% of samples (minimum 3 samples) were retained to ensure statistical stability; (4) ASVs with coefficient of variation below 0.1 were filtered out to focus on taxa with meaningful abundance variation; and (5) a pseudocount of 1 was added to all abundance values to stabilize log-ratio transformations and avoid issues with zero values. Then, we quantified the proportionality of change (Rho) between pairs of ASVs using the propr package (Quinn et al., 2017). This package computes centered log-ratio (CLR) transformations to robustly assess association patterns in compositional data while avoiding spurious correlations inherent to relative abundance data. These results were filtered to achieve a false discovery rate (FDR) below 5% using permutation testing (*n* = 100 permutations) with the updateCutoffs function.

We focused on genera containing at least 10 closely related ASVs, defined as having a maximum nucleotide divergence of 5 nucleotides, following the methodology of Auladell et al. (2022). This threshold was selected because: (1) it ensures sufficient statistical power for proportionality analysis and linear modeling; (2) it focuses on ecologically relevant closely-related taxa that are more likely to share similar niche requirements; and (3) for our V4-V5 hypervariable region of the 16S rRNA gene (~412 bp), 5 nucleotide divergence corresponds to approximately 98.7% sequence identity, indicating close phylogenetic relatedness consistent with potential ecological coherence within genera.

Within each genus, we evaluated the Rho value between pairs of ASVs as a proxy for niche similarity and compared it against nucleotide divergence among ASVs to identify trends in niche relatedness. For each genus meeting our criteria, we fitted a linear model of the form: Rho ~ nucleotide_divergence, using ordinary least squares regression. Model assumptions were verified through residual analysis. To account for multiple testing across genera, we applied FDR correction (Benjamini–Hochberg method) to the *p*-values from individual genus models, with statistical significance defined as FDR-adjusted *p* < 0.05.

### Community assembly processes

To disentangle the relative contributions of stochastic and deterministic processes in shaping microbial community assembly, we applied the phylogenetic bin-based null model analysis implemented in the iCAMP package (Ning et al., 2020). This approach clusters ASVs into distinct phylogenetic bins, enabling resolution of community dynamics across evolutionary scales. For each bin, assembly mechanisms were inferred through null model analysis of phylogenetic and taxonomic β-diversity. Specifically, the beta nearest taxon index (βNRI) was used to assess phylogenetic structure, while the modified Raup-Crick metric (RC) quantified taxonomic turnover. Based on these indices, community assembly was classified into five processes: homogeneous selection (HoS) and heterogeneous selection (HeS) (deterministic processes); and dispersal limitation (DL), homogenizing dispersal (HD) and ecological drift (DR) (stochastic processes). To scale these bin-level inferences to the entire community, the relative influence of each process was calculated by weighting the contribution of each bin by its relative abundance. This allowed estimation of the overall importance of each assembly process at the community level.

### Associations among assembly processes and environmental variables

To examine the relationship between community assembly processes and environmental drivers we followed an approached previousl reported (Ning et al., 2024). First, the relative importance of each assembly process estimated by iCAMP was transformed using the centered log-ratio (clr) transformation to account for the compositional nature of the data. Environmental variables were log-transformed (after adding 1 to avoid zeros) to improve normality. For each pairwise combination of a clr-transformed assembly process and a log-transformed environmental variable, we fitted a linear model using a Monte Carlo cross-validation scheme: 90% of samples were randomly selected as training data, and the model's predictive performance was evaluated on the remaining 10% (repeated 1,000 times). The cross-validated coefficient of determination (*R*^2^CV) was used as a measure of association strength. Statistical significance was assessed via permutation testing (999 randomizations of the response variable), yielding empirical *p*-values. Benjamini–Hochberg false discovery rate (FDR) correction was applied to control for multiple comparisons across all process-environment pairs. Associations were considered significant if they had FDR-adjusted *p* < 0.05 and *R*^2^CV > 0.01.

We further evaluated multicollinearity among environmental variables using variance inflation factors (VIF). As expected in acid mine drainage systems, where metal concentrations and pH are geochemically linked, several variables exhibited high intercorrelation, with VIFs exceeding 5 for pH (7.97), Cu (5.32), Cd (7.58), Mn (21.27), and Zn (40.66). Because our analysis tested one environmental variable at a time in bivariate models, collinearity did not affect the statistical estimation of each association. However, we recognize that significant associations may represent the influence of correlated suites of environmental drivers rather than the unique effect of a single variable. For this reason, all variables were retained to preserve ecological interpretability.

### Environmental parameter measurements

Water samples were immediately filtered on-site through 0.45 μm polyethersulfone syringe filters (50 mm, Anpu, Shanghai) into pre-cleaned 50 mL polypropylene centrifuge tubes (Shuohua, Zhejiang). Each aliquot was acidified to pH < 2 with ultrapure concentrated HNO3 (Aladdin, Shanghai) and stored at 4 °C until analysis. Temperature, pH, oxidation-reduction potential (ORP), dissolved oxygen (DO), and electrical conductivity were recorded at the sampling point with a portable Hach HQ-series multiparameter probe (CO, USA). In the laboratory, dissolved trace metals were quantified by ICP-MS (iCAP RQ, Thermo Fisher Scientific, USA), while ${\text{SO}}_{4}^{2-}$ was determined by ion chromatography (AQuion, Thermo Fisher Scientific, USA).

## Results and discussion

### Physicochemical landscape of the acid mine drainage system

**Figure 2:**
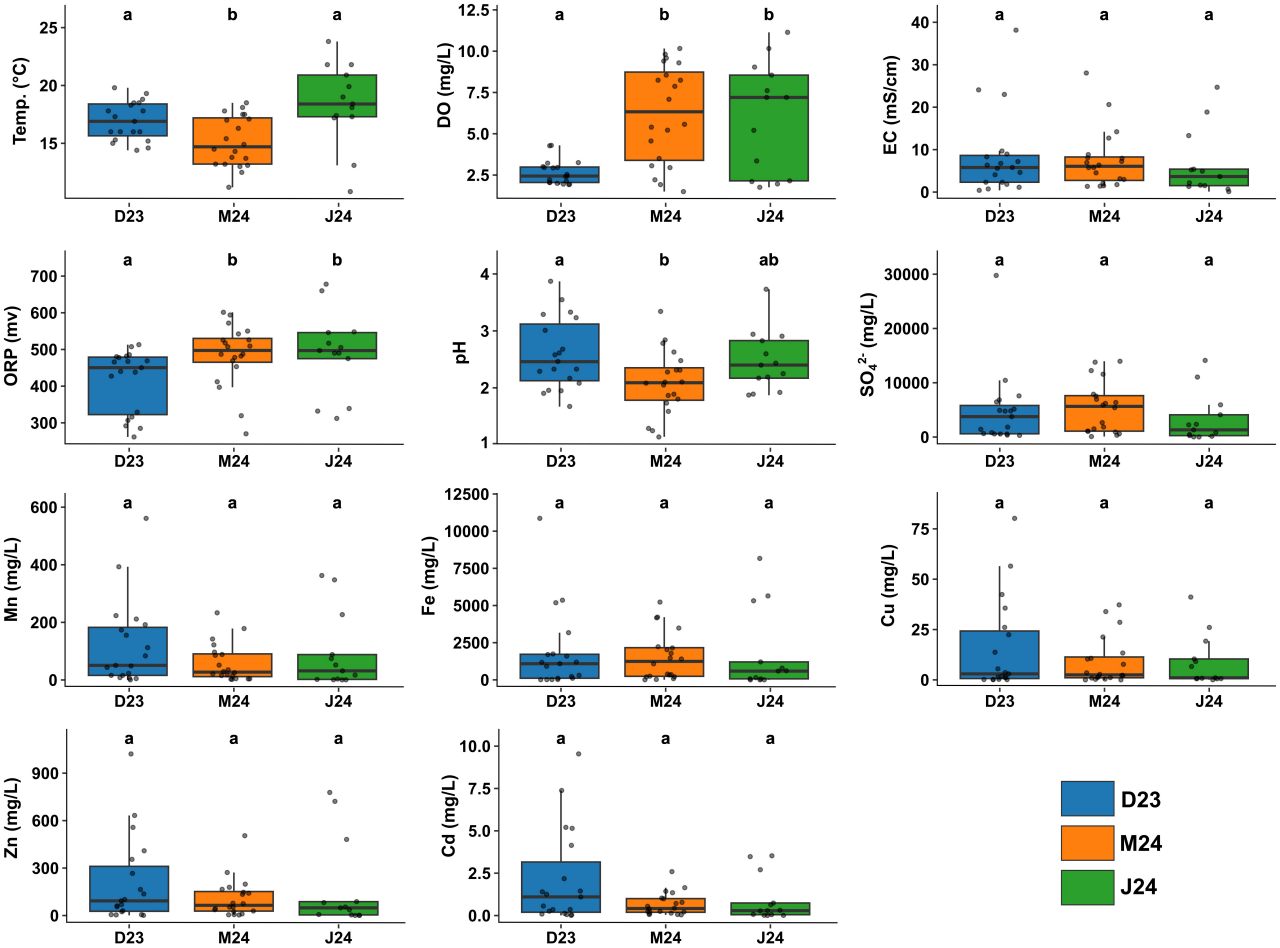
Physicochemical parameters of the acidified samples (pH < 4) collected during the three sampling events. Significant differences among sampling events were assessed using Kruskal–Wallis tests followed by Dunn's *post-hoc* comparisons with Bonferroni correction. Different letters above boxplots indicate significant differences at *p* < 0.05. For the complete dataset including all samples irrespective of pH, see Supplementary Figure S1.

The studied mining area in Zhejiang province, China, sampled across 45 sites, presented extreme physicochemical conditions characteristic of acid mine drainage (AMD) environments (Figure 1). Water samples collected across three sampling events (December 2023, March 2024, June 2024) were consistently acidic with a median pH of 2.5 and substantial dissolved metal loads (Figure 2, Supplementary Figure S1). Iron dominated with a median of 1,365 mg/L and maximum exceeding 10,000 mg/L, followed by manganese (median of 110 mg/L and maximum of 560 mg/L), while zinc, copper, and cadmium occurred at lower levels. Electrical conductivity and sulfate concentrations were persistently elevated, with sulfate reaching a median of 1,750 mg/L, and oxidation-reduction potential remained high at a median of 470 mV (Figure 2, Supplementary Figure S1). These conditions collectively align with typical AMD environments where sulfide mineral oxidation generates acidity and mobilizes metals (Sánchez España et al., 2005; Huang et al., 2021).

Spatial heterogeneity, rather than temporal dynamics, primarily drove physicochemical variation. While December 2023 included sites with broader pH ranges (Figure 1, Supplementary Figure S1), subsequent sampling events focused on the most acidic sites. Among acidic samples (pH < 4), only dissolved oxygen showed significant temporal variation, with lower concentrations in December 2023 (~2.5 mg/L) than in March and June 2024 (7–8 mg/L; Figure 2), while metal concentrations remained stable. Temporal differences became statistically non-significant when restricted to consistently monitored sites (*n* = 5; Supplementary Figure S2), confirming spatial heterogeneity as the primary driver. This pattern is consistent with findings from other AMD systems where spatial variability in mineral composition, hydrological flow paths, and microbial activity create distinct geochemical microenvironments that persist over time (Méndez-García et al., 2014), generating diverse ecological niches that could support specialized microbial communities adapted to specific physicochemical conditions.

### pH as the primary driver of microbial community structure

**Figure 3:**
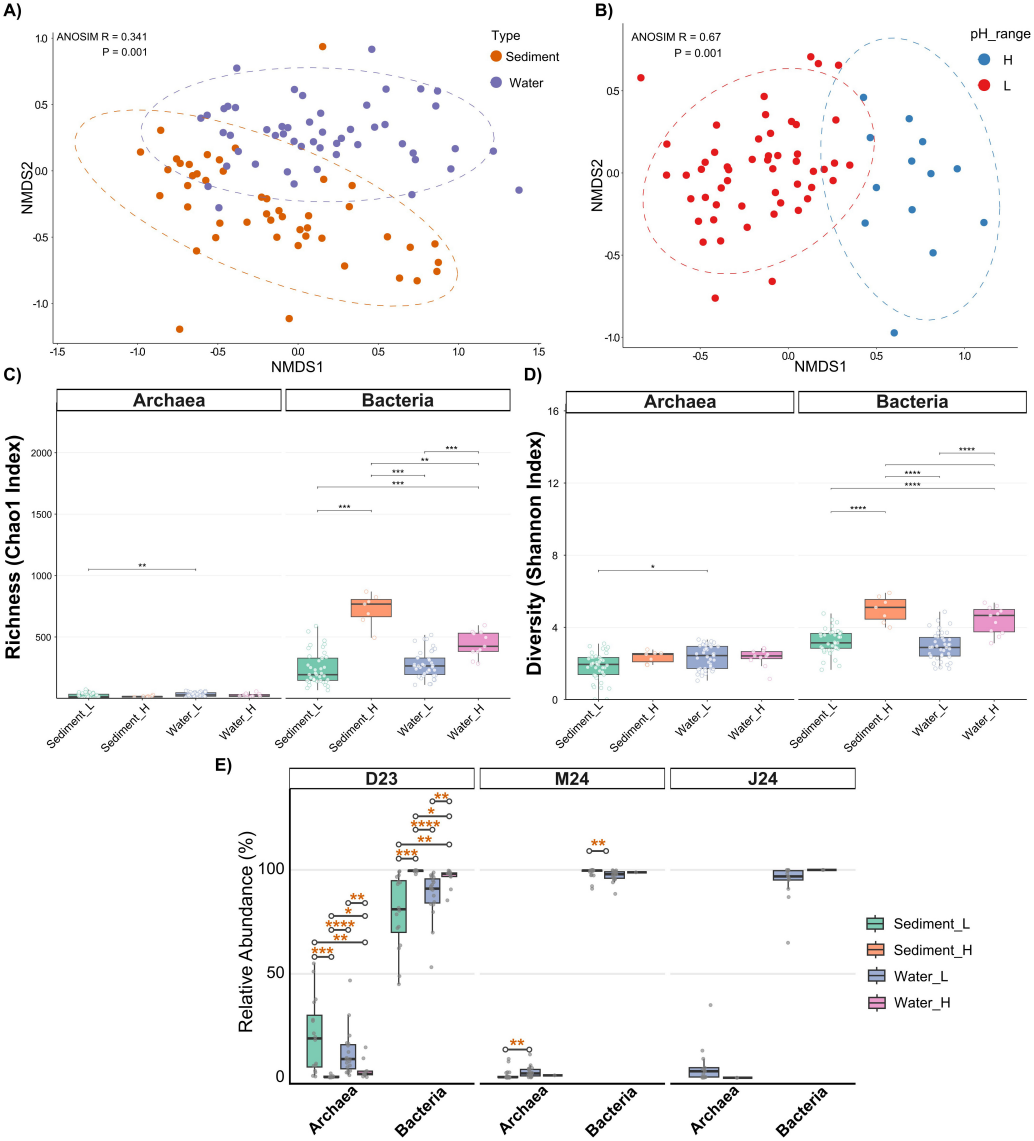
Non-metric multidimensional scaling (NMDS) ordination based on microbial community composition, with sites colored by **(A)** sample type and **(B)** pH level. Ellipses represent 95% confidence intervals for each group. Boxplots show **(C)** alpha richness (Chao1 Index) and **(D)** alpha diversity (Shannon index) across samples. **(E)** Relative abundance of Archaea and Bacteria across samples, stratified by sampling time point. Significant differences were assessed with Wilcoxon rank-sum tests with Benjamini–Hochberg FDR correction: ****p.adj < 0.0001; ***p.adj < 0.001; **p.adj < 0.01; *p.adj < 0.05.

The extreme environmental conditions, particularly the strong pH gradient, exerted a profound influence on the microbial community structure. Non-metric multidimensional scaling (NMDS) analysis revealed pH as the most significant factor structuring microbial assemblages (ANOSIM R = 0.67, *p* = 0.001) (Figure 3A), consistent with other studies indicating pH as primer environmental driver of microbial community composition in acidic environments (Kuang et al., 2013). Sample type (water vs. sediment) also emerged as a significant, albeit secondary, driver of community structure (ANOSIM R = 0.34, *p* = 0.001) (Figure 3B), indicating that distinct microbial communities colonize the planktonic and benthic habitats within the AMD system. Temporal variations, however, had a minimal impact on overall community composition, especially when considering only the consistently monitored sites (Supplementary Figure S3).

The strong structuring effect of pH was also reflected in the alpha diversity patterns (Figures 3C, D). Bacterial communities, which constituted the vast majority of the microbiome, exhibited significantly lower richness and diversity in low-pH samples than in higher-pH samples (*p* < 0.001) (Figures 3C, D), reflecting intense selective pressure imposed by acidity that severely limits colonizing taxa (Carlier et al., 2020). Previous studies of acidic systems have consistently reported greater bacterial richness and diversity in sediments compared to the overlying water column (García-Moyano et al., 2012; Carlier et al., 2020). In our dataset, however, bacterial alpha diversity (i.e., richness and diversity) did not differ significantly between these habitats (*p* > 0.05), except at higher pH sites where sediment samples exhibited significantly elevated richness compared to water (*p* < 0.01) (Figures 3C, D). Archaeal communities comprised a minor fraction of total abundance (< 10% in most samples; Figure 3E), with an exception in December 2023 when archaeal abundance exceeded 50% in some low-pH sediment samples (Figure 3E). This temporary increase likely reflects the lower dissolved oxygen levels observed during that sampling period, as some archaeal acidophiles are especially tolerant to low-oxygen conditions (Distaso et al., 2020; Neira et al., 2021). Archaeal richness and diversity remained uniformly low across all conditions (Figures 3C, D), with only minor increases observed in low-pH water samples compared to sediment samples. Beyond pH and habitat, environmental dissimilarity correlated with community dissimilarity in low-pH waters (Supplementary Figure S4), indicating additional fine-scale physicochemical controls.

### Persistent abundant taxa in acidic environments

**Figure 4:**
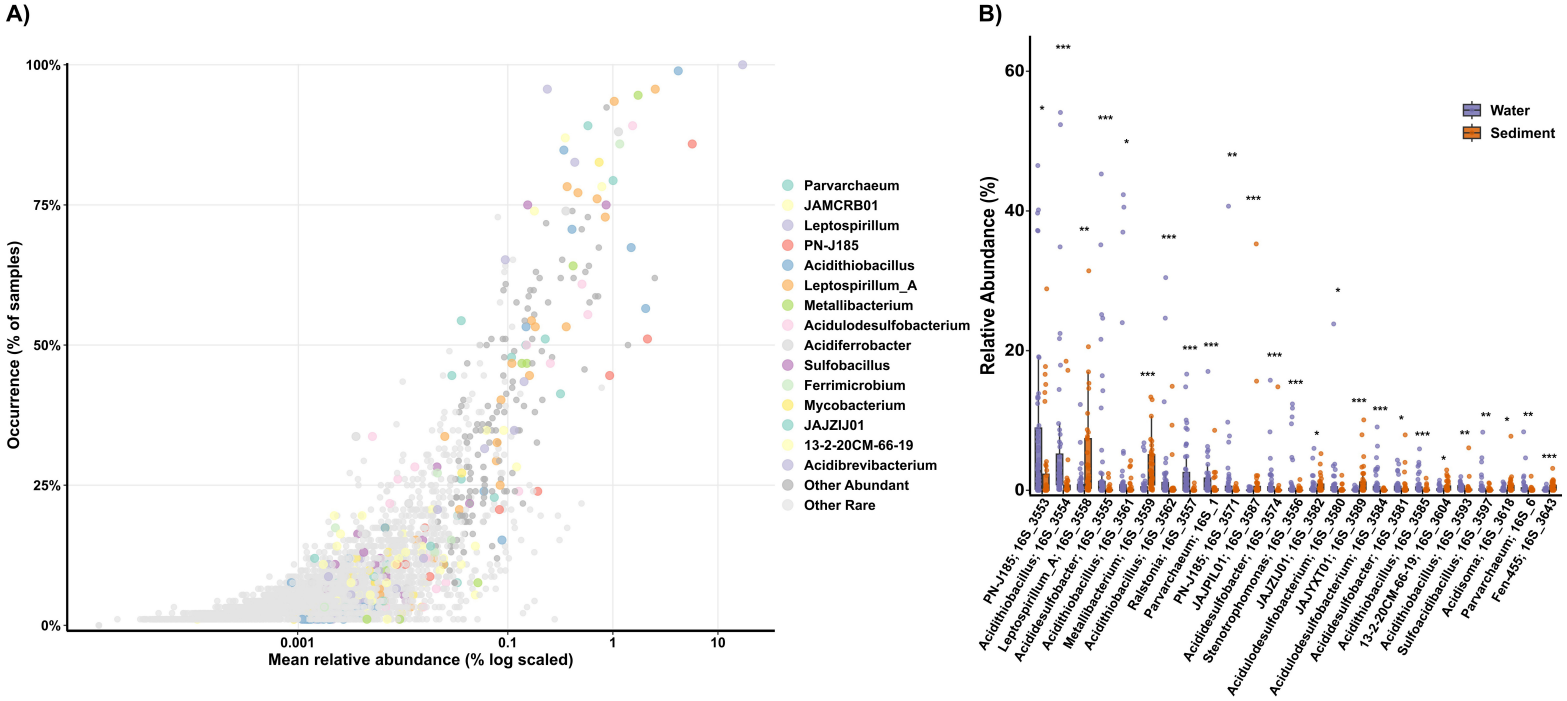
Distribution and habitat specificity of ASVs in acidic samples. **(A)** ASV occurrence vs. mean relative abundance. Dot color indicates taxonomic affiliation. **(B)** Relative abundance of the top 25 ASVs with significant differences between water and sediment samples. Significant differences were assessed with Wilcoxon rank-sum tests with Benjamini–Hochberg FDR correction: ***p.adj < 0.001; **p.adj < 0.01; *p.adj < 0.05.

Taxonomic profiles aligned with the pH gradient, with low-pH samples enriched in classical acidophiles (e.g., *Leptospirillum, Acidithiobacillus, Metallibacterium*), while higher-pH sites were enriched in neutrophilic iron oxidizers (e.g., *Sideroxyarcus, Gallionella, Leptothrix, Acidovorax*) and *Burkholderiales* ASVs (Supplementary Figure S5). Given that the AMD system was predominantly acidic, we further focused on characterizing the persistent abundant microbial taxa thriving under these conditions. Analysis of the 3,374 Amplicon Sequence Variants (ASVs) detected in acidic samples revealed that the community was dominated by a small number of widespread and abundant taxa, while the majority of ASVs were rare and sporadically distributed (Figure 4A). Strikingly, 78% of ASVs had narrow occurrence ( ≤ 10% of samples), while only 23 ASVs exhibited persistent abundance (≥75% occurrence and ≥1% relative abundance in at least one sample). These persistent abundant taxa included well-known acidophiles such as *Leptospirillum, Acidithiobacillus, Ferrimicrobium*, and *Parvarchaeum*. Additional persistent taxa were affiliated with *Acidulodesulfobacterium, Acidibrevibacterium, Metallibacterium, Acidiferrobacter*, PN-J185 (*Ferrovum*-like), *Sulfobacillus*, and *Leptospirillum_A* (five ASVs; Figure 4A). several uncultured lineages including the archaeal JAMCRB01 and bacterial genera JAJZIJ01 and 13-2-20CM-66-19 were among broadly distributed abundant taxa. While *Leptospirillum* and *Acidithiobacillus* have been frequently reported in AMD systems (Johnson and Hallberg, 2003), others such as *Acidulodesulfobacterium, Acidibrevibacterium*, and especially the aforementioned uncultured genera have been rarely documented in AMDs, expanding the known diversity of key acidophiles in these systems. Additionally, 167 ASVs presented intermediate occurrence patterns (>10% and < 75% of samples) with abundance above 1% in at least one sample. This group included genera such as *Ferroplasma, Acidisoma, Acididesulfobacter*, and uncultured genera including JAJYXT01, JAKAFX01, JAMDCX01, and REEB76. Notably, *Ferroplasma* was one of the few genera to show significant temporal variation, with a higher abundance in the December 2023 samples (Supplementary Figure S3). We also identified 160 ASVs as Conditionally Rare Taxa (CRT), which typically remain rare in most samples but occasionally become abundant. *Thiomonas* was the genus with the highest number of ASVs considered CRT. Several *Thiomonas* isolates can oxidize iron under mildly acidophilic to neutrophilic conditions (Akob et al., 2020), suggesting that they could bloom in samples with moderate acidity. Other CRTs were associated with *Acidocella, Acidiphilium, Ferroacidibacillus, Acidifodinimicrobium, Acidithrix*, and *Sulfoacidibacillus*, all of which can thrive heterotrophically in micro-oxic or anaerobic environments while reducing ferric iron. Thus, these CRTs may represent taxa adapted to specific microhabitats or environmental conditions unique to particular samples within the studied system, potentially playing essential roles under specific circumstances.

### Habitat-based partitioning between water and sediment

Analysis of dominant acidophiles revealed distinct habitat partitioning patterns (Figure 4B). Several taxa demonstrated generalist distributions with no significant abundance differences between water and sediment samples (*p* > 0.05; Supplementary Figure S6). These included ASVs affiliated with *Leptospirillum, Acidulodesulfobacterium*, and *Ferrimicrobium*, as well as most *Leptospirillum_A* ASVs (Supplementary Figure S6). In contrast, strong sediment preferences (*p* < 0.05–0.001) were observed for *Metallibacterium* and multiple uncultured genera (JAJPIL01, JAJYXT01, 13-2-20CM-66-19), while water samples were enriched in PN-J185, *Acidithiobacillus, Acididesulfobacter* and *Parvarchaeum* ASVs (*p* < 0.05–0.001; Figure 4B). This habitat-specific partitioning suggests differentiation between microbial taxa with planktonic lifestyle preferences and those adapted to sediment matrices. Planktonic taxa may require dissolved substrates or oxygen availability in the water column, while sediment-adapted taxa thrive in more heterogeneous chemical environments with potential anaerobic micro-niches (García-Moyano et al., 2012).

### Metal tolerance specialists and niche segregation among iron oxidizers

**Figure 5:**
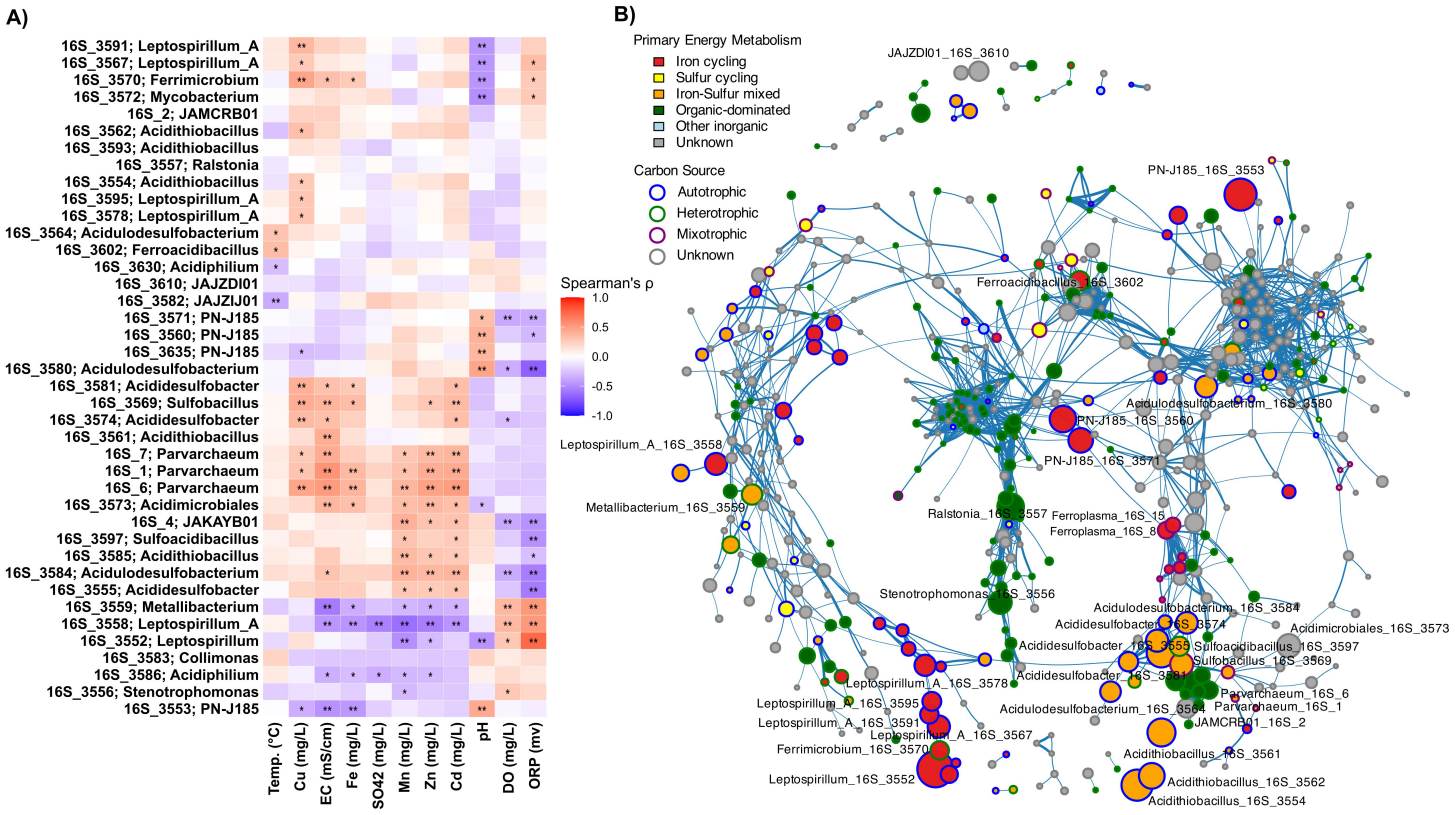
Correlation and co-occurrence patterns among ASVs. **(A)** Spearman correlations between dominant ASVs and physicochemical parameters in low-pH water samples (pH < 4). Significant correlations are indicated by *p < 0.05 and **p < 0.01. Clustering highlights co-varying ASVs and environmental factors, revealing fine-scale niche partitioning. **(B)** Network of significant positive correlations among ASVs across all samples (Spearman r ≥ 0.6, FDR-adjusted *p* < 0.01). Node colors indicate predominant energy metabolism (inner) and carbon source utilization strategy (outer ring), illustrating modular co-occurrence patterns among acidophiles.

Within low-pH waters, co-occurrence networks and abundance-environment correlations revealed fine-scale niche separation (Figure 5). One distinct cluster of ASVs showed significant positive correlations with metal concentrations and electrical conductivity, including multiple *Parvarchaeum* ASVs, several *Acididesulfobacter* variants, and an ASV affiliated with *Sulfobacillus* (*p* < 0.05–0.01; Figure 5A). Additional taxa including *Acidulodesulfobacterium, Acididesulfobacter, Sulfoacidibacillus*, and *JAKAYB01* were similarly correlated with high metal loads under lower redox conditions (*p* < 0.05–0.01; Figure 5A). This group shares metabolic capacity for oxidizing both ferrous iron and sulfur compounds. The archaeal acidophiles *Parvarchaeum* and *JAKAYB01* are exceptions, as their metabolism remains unclear but may be more carbon-focused. These taxa also clustered together in the co-occurrence network, displaying strong positive correlations among them (Spearman's ρ > 0.6, *p* < 0.01; Figure 5B).

Despite shared iron-oxidation capacity, iron oxidizers segregated into distinct niches. One group composed of *Leptospirillum_A* variants (ASV_3591 and ASV_3567), *Ferrimicrobium*, and *Leptospirillum* was strongly associated with extremely low pH, elevated copper concentrations, and elevated ORP levels (Figure 5A). In contrast, the *Ferrovum*-like iron oxidizer PN-J185 was associated with increasing pH and lower ORP. It clustered separately from other acidophilic iron oxidizers and showed stronger associations with the neutrophilic iron oxidizer *Gallionella* (ASV_3954; Spearman's ρ > 0.6, *p* < 0.01; Figure 5B). These findings are consistent with reported data showing that *Ferrovum* spp. would tolerate broader pH ranges than obligate acidophiles (Ullrich et al., 2016; Grettenberger et al., 2020). A distinct *Leptospirillum_A* variant (ASV_3558) was associated with higher dissolved O_2_ and ORP but lower metal concentrations (*p* < 0.01; Figure 5A). This cluster also included two ASVs associated with *Metallibacterium* (Figure 5A), a metabolically versatile genus capable of oxidizing various organic and inorganic compounds such as tetrathionate and hydrogen while reducing ferric iron; traits that could support adaptation to fluctuating redox conditions (Bartsch et al., 2017). Another separate cluster comprised several ASVs classified as *Ferroplasma* (Spearman's ρ > 0.6, *p* < 0.01; Figure 5B), while the iron oxidizer *Ferroacidibacillus* occupied an isolated niche with minimal correlation to other iron oxidizers and weak associations with measured physicochemical parameters except for a moderate positive correlation with temperature (*p* < 0.05; Figure 5A). Notably, *Acidithiobacillus* ASVs, among the most abundant microorganisms in the samples, showed minimal correlations with other taxa and weak associations with most physicochemical parameters (Figures 5A, B). This pattern may reflect their exceptional metabolic versatility, including capabilities for both oxidation and reduction of iron and sulfur compounds. This versatility enables persistence across diverse environmental conditions without requiring specific partnerships or narrow environmental constraints. This metabolic flexibility likely contributes to their widespread distribution and abundance in AMD systems globally (Quatrini and Johnson, 2019).

Together, this intricate pattern of correlations demonstrates that even within the highly selective acidic environment, microbial niches are further segregated based on specific metal concentrations, redox potential, oxygen availability, and pH gradients. The analysis reveals a hierarchical niche structure. Broad habitat preferences (water vs. sediment) are further refined by specific physicochemical tolerances and metabolic specializations. This refinement results in distinct ecological clusters that reflect both phylogenetic constraints and environmental adaptation.

### Microdiversity patterns and ecological significance

To investigate niche differentiation at finer resolution, we analyzed effective microdiversity patterns in OTUs with >5,000 total reads, retaining 97 OTUs that represented a substantial fraction of total community abundance. Effective microdiversity ranged from virtually no variation (~1 effective ASV) to high microdiversity (>6 effective ASVs; Supplementary Figure S7). Of these, 49 OTUs exhibited fewer than two effective ASVs, while 46 OTUs exceeded this threshold. Examples of OTUs with high effective microdiversity included OTU_3877 (JAKAOD01), OTU_3567 (*Leptospirillum_A*), and OTU_3580 (*Acidulodesulfobacterium*). Interestingly, microdiversity was not uniform within genera. While one *Acidulodesulfobacterium* OTU contained very high effective microdiversity (>4), other OTUs assigned to this genus had very limited effective microdiversity (Supplementary Figure S7). Similar patterns were observed for the *Leptospirillum_A* genus, with several OTUs displaying high microdiversity (>3.5 ASVs) while others remained low (< 1.5 ASVs) (Supplementary Figure S7). These results suggest that the potential for intra-species variation is not uniform across a genus, possibly reflecting different evolutionary histories, selective pressures, or functional constraints among lineages within the same genus (Koeppel and Wu, 2013). Importantly, overall patterns of effective microdiversity were not influenced by OTU abundance (*p* > 0.05) (Supplementary Figure S9), and no differences in effective microdiversity were detected based on habitat type or pH level (*p* > 0.05) (Supplementary Figure S9). These findings suggest that the observed variation represents stable evolutionary adaptations rather than transient demographic effects.

We then examined the ecological significance of this microdiversity by relating it to community stability. OTUs with higher effective microdiversity (≥2 ASVs) were significantly more persistent (i.e., found in a larger proportion of samples) than those with low microdiversity (< 2 ASVs) (*p* < 0.0001; Supplementary Figure S10). This positive association suggests that a greater pool of closely related genotypes might contribute to a taxon's persistence across the heterogeneous landscape of the AMD system. This finding aligns with previous studies demonstrating that microdiversity can enhance community stability and resilience in the face of environmental fluctuations (García-García et al., 2019). However, environmental stress appears to modulate this stabilizing effect. In samples with low pH or high iron concentrations, OTUs exhibited greater abundance variability than OTUs in less extreme conditions, particularly those OTUs with high microdiversity (*p* < 0.001; Supplementary Figure S10). These results contrast with other reports that demonstrated higher microdiversity not only enhances persistence, as also observed in our study, but also promotes lower variability. This pattern contrasts with studies in less extreme environments such as freshwater (Linz et al., 2017; García-García et al., 2019; Okazaki et al., 2021), drinking water (He et al., 2025), or seawater (Needham et al., 2017; Mende et al., 2019), where higher microdiversity not only enhances persistence but also promotes lower variability. Our findings suggest that while microdiversity generally promotes persistence across environmental gradients, extreme physicochemical pressures can amplify abundance fluctuations even within microdiversity-rich lineages. This creates a complex, context-dependent relationship between diversity, stability, and environmental stress. Several mechanisms may explain these observed patterns. First, extreme stress may reduce effective population sizes to levels where demographic stochasticity overwhelms stabilizing selection (Shade et al., 2012), which can lead to amplified abundance fluctuations despite niche differentiation among ASVs. Second, under extreme conditions, the stress-gradient hypothesis (SGH) may apply, this is, facilitative interactions become more important than competitive interactions (Bertness and Callaway, 1994; Hammarlund and Harcombe, 2019), and shifts in dominant facilitators could trigger cascading abundance changes across multiple ASVs within an OTU. Third, the harsh physicochemical conditions may create temporal windows where only specific genotypes within a microdiversity pool can persist (Yachi and Loreau, 1999; Lennon and Jones, 2011), leading to sequential turnover of ASVs rather than stable coexistence.

### Fine-scale habitat partitioning among closely related genotypes

**Figure 6:**
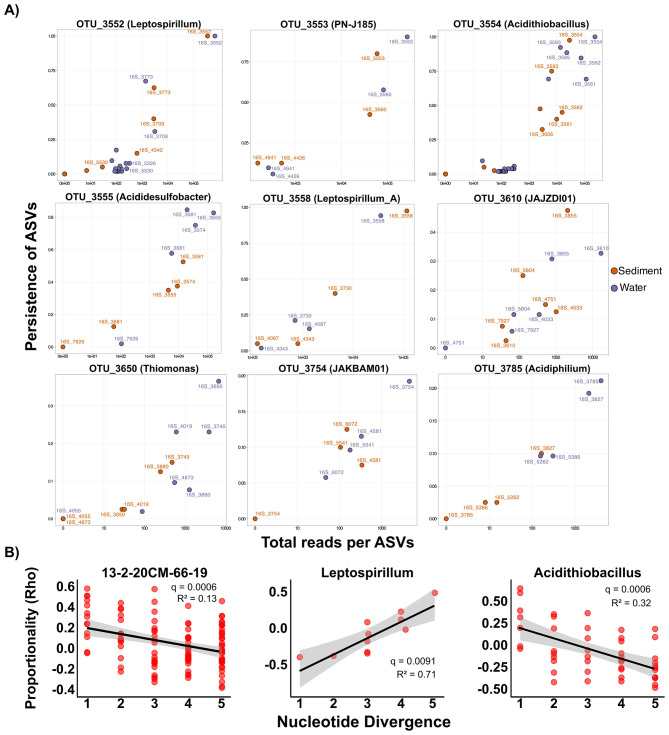
Niche and persistence patterns of ASVs. **(A)** Scatter plots of log-transformed overall relative abundance (read counts) and persistence for ASVs within OTU in sediment (orange) and water (purple) samples. Each dot represents a single ASV, and the most abundant ASVs across the studied AMD system are labeled with the ASV-IDs. **(B)** Relationships between pairwise nucleotide divergence and niche similarity (Rho) for genera showing significant correlations (FDR-adjusted *p* < 0.05). Results for all analyzed genera are provided in Supplementary Figure S12.

Analysis of ASV distributions within individual OTUs provided evidence of fine-scale habitat partitioning among closely related genotypes (Figure 6A). Several OTUs exhibited generalist distributions with comparable abundance between water and sediment (e.g., *Leptospirillum*, PN-J185, *Leptospirillum_A*), while others showed pronounced habitat specificity. Notable examples include ASV_3585 (*Acidithiobacillus*), abundant in water (>80% occurrence) but rare in sediments (< 10%), as well as other OTUs affiliated to *Acidiphilium*, JAKBAM01, *Thiomonas*, where different ASVs dominated water vs. sediment (Figure 6A). Environmental gradients also structured ASV distributions: OTU_3612 (*Hydrogenophaga*) showed pH-dependent partitioning, while OTU_3610 (JAJZDI01) exhibited iron concentration-dependent ASV turnover (Supplementary Figure S11). These findings demonstrate that even minimal genetic divergence between ASVs can translate into significant ecological differentiation, allowing closely related genotypes to exploit distinct niches within the broader AMD environment. Studies across diverse ecosystems have also shown that minimal genetic divergence enables niche differentiation (Auladell et al., 2022; Fu et al., 2023), and our results extend this principle to the sub-species level in extreme environments where competitive exclusion is traditionally predicted to dominate due to strong environmental filtering and reduced niche space (Nemergut et al., 2013; Carlier et al., 2020).

To explore how niche similarity relates to phylogenetic distance within key genera, we conducted Rho proportionality analysis, which quantifies proportional change between ASV pairs as a proxy for niche overlap. Among the evaluated genera (those with at least 10 distinct ASVs), *Acidithiobacillus* and 13-2-20CM-66-19 displayed significant decreases in Rho proportionality with increasing nucleotide divergence (adjusted *p* < 0.001; Figure 6B, Supplementary Figure S12). This negative relationship indicates that closely related ASVs occupy more similar ecological niches, while divergent ASVs show stronger niche differentiation. Such patterns are consistent with phylogenetic clustering observed in other ecosystems (Salter et al., 2015; Tromas et al., 2018) and in AMDs (Liang et al., 2017). Significantly, *Leptospirillum* showed an opposite trend, with niche similarity increasing with genetic divergence (adjusted *p* < 0.001), suggesting that diversification within this genus may enable complementary niche exploitation rather than competition. These contrasting patterns demonstrate that deterministic filtering and adaptive radiation can operate simultaneously within the same AMD community, depending on the lineage. Nevertheless, the predominant pattern across most analyzed genera was no significant relationship between phylogenetic distance and niche similarity (adjusted *p* > 0.05; Supplementary Figure S12). The lack of consistent phylogenetic signal in niche similarity could be attributed to rapid evolutionary responses under strong selection pressure, horizontal gene transfer obscuring phylogenetic relationships in functional traits, or limitations of the 16S rRNA gene in capturing genomic changes that impact niche distribution (VanInsberghe et al., 2020).

In water samples, where a broader range of environmental parameters were measured, niche similarity generally showed weak relationships with environmental distance, except for *Nobosphingobium* and *Thiomonas*, where niche similarity increased with rising environmental distance (Supplementary Figure S13). This positive association points to potential facilitation or complementary resource use under fluctuating conditions (Chase and Leibold, 2003), consistent with the idea that cooperative interactions may be particularly important for persistence in stressful environments such as AMD systems (Paerl and Pinckney, 1996).

Overall, these findings suggest that AMD communities exhibit lineage-specific strategies rather than conforming to a single pattern of phylogenetic-niche relationships. Both convergence among close relatives and divergence among distant relatives contribute to coexistence, underscoring the complexity of assembly mechanisms in extreme environments.

### Community assembly processes and environmental drivers

**Figure 7:**
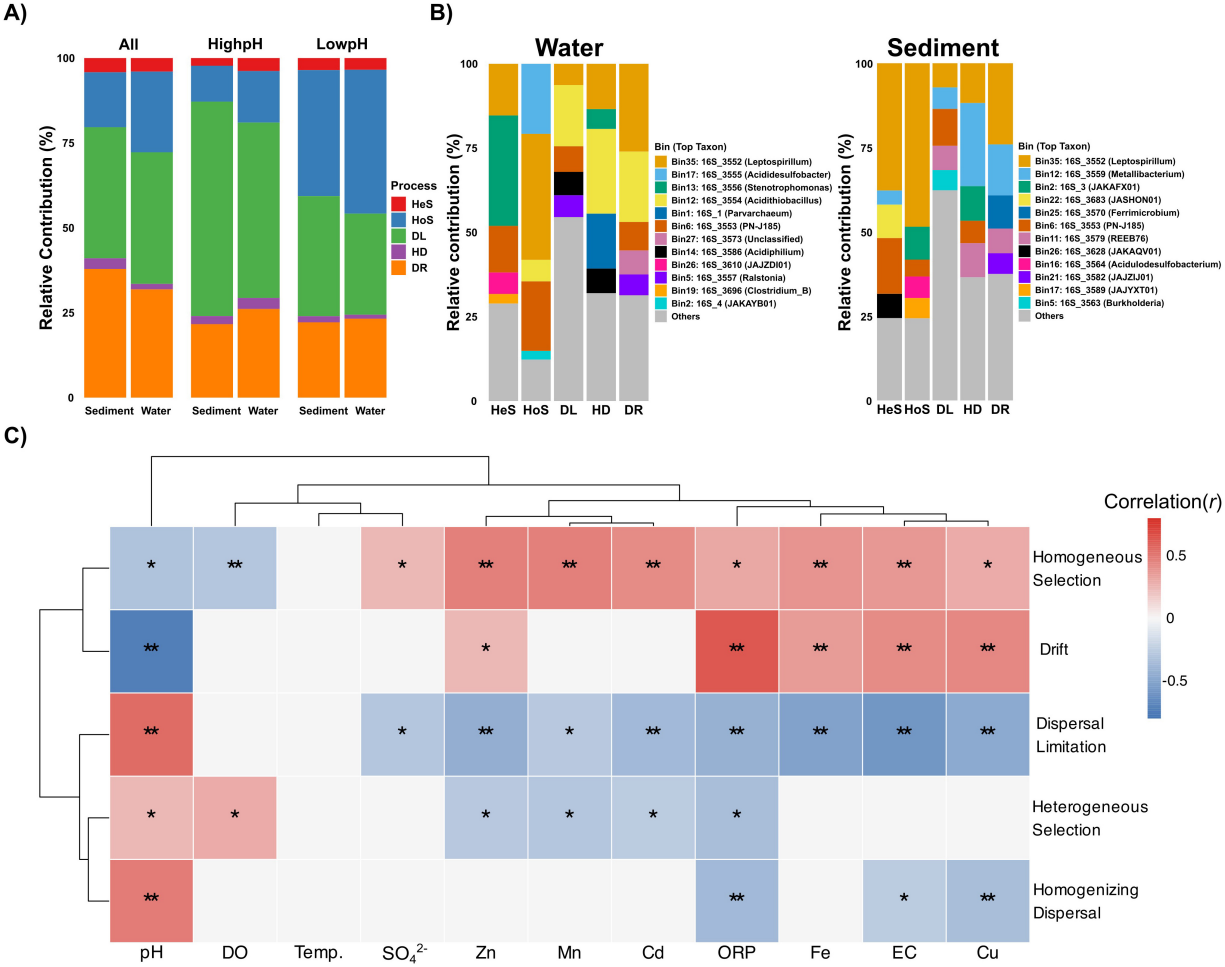
Microbial community assembly processes and environmental drivers. **(A)** Relative contributions of ecological processes across habitats and pH conditions. **(B)** Relative abundance of different genera governed by the distinct assembly processes (HeS and HoS deterministic processes, while DL, HD and DR as stochastic processes) for water (left) and sediment (right) samples. **(C)** Correlation between physicochemical parameters and ecological assembly processes. Differences in process proportions between groups were tested using Wilcoxon rank-sum tests with Benjamini–Hochberg FDR correction. Significance is indicated by symbols: **p ≤ 0.01; *p ≤ 0.05.

To understand the fundamental forces shaping these microbial communities, we quantified ecological assembly processes using iCAMP (phylogeny-informed null modeling) (Ning et al., 2020). Across the system, dispersal limitation was the dominant process, accounting for 39% of community turnover in both water and sediment (Figure 7A). This indicates that restricted microbial movement between sites is a primary constraint on community composition. Ecological drift also played a substantial role, contributing more in sediments (38%) than in water (32%; *p* < 0.001), suggesting that random demographic fluctuations more strongly influence benthic communities. Homogeneous selection was the next most important process, particularly in the water column (24% vs. 16% in sediments; *p* < 0.001), whereas heterogeneous selection (< 5%) and homogenizing dispersal (< 3%) were consistently minor (Figure 7A).

When stratified by pH conditions, clear shifts emerged. Under extreme acidity (low pH), deterministic processes became dominant, especially in water, where homogeneous selection accounted for nearly 40% of assembly. In contrast, dispersal limitation was strongest at higher pH, reaching 50% in water and 70% in sediments (*p* < 0.001) (Figure 7A). These patterns show that intense environmental filtering drives community assembly under harsh chemical stress, whereas spatial barriers govern assembly in less stressful habitats. Importantly, even under extreme acidity, stochastic processes such as drift contributed substantially, highlighting that randomness in population dynamics persists and interacts with strong deterministic filtering. The assembly patterns in acidic waters from our study mine resemble those found in lakes with varying AMD contamination, where homogeneous selection was also dominant followed by drift and dispersal limitation (She et al., 2023). Similarly, a study on groundwater habitats showed a shift from stochastic to more deterministic processes with increasing environmental stress, primarily related to pH and metal concentrations (Ning et al., 2024).

We also examined the impact of the assembly processes on the different microbial groups in the acidic samples. The observed OTUs were divided into 62 (water samples) and 53 (sediment samples) phylogenetic groups (bins). Dispersal limitation dominated the majority of taxonomic groups across all sample types, governing 70% of bins in sediments and 83% in water samples (Supplementary Figure S14). Homogeneous selection governed 20% of bins in sediment and 11% in water, while drift processes were more prominent in sediment environments, controlling 7.5% of bins compared to only 4.3% in water samples. Similar observations were reported in a meta-analysis of global AMD sites using the same iCAMP approach, but in that study, all 26 phylogenetic bins generated were dominated by dispersal limitation (Liu et al., 2025). Abundance-weighted phylogenetic bin analysis revealed that for each process, only five bins accounted for more than 50% of the total contribution, indicating that dominant organisms play disproportionate roles in shaping overall community dynamics. Dispersal limitation was an exception, being represented across a wide variety of bins (Figure 7B). A *Leptospirillum*-associated bin was the most significant contributor to drift in both sediment and water samples (Figure 7A), with also important contributions from *Metallibacterium* in sediments and *Acidithiobacillus* in water. The dominant bins governed by homogeneous selection in sediments were associated with *Leptospirillum*, JAKAFX01, *Acidulodesulfobacterium*, and JAJYXT01, while in water, this process was mainly attributed to *Leptospirillum*, PN-J185, and *Acididesulfobacter* (Figure 7B). This taxon-specific variation in assembly processes highlights the importance of considering microbial identity when studying community assembly, as different taxa may be subject to different ecological constraints even within the same environment (Stegen et al., 2013).

Environmental gradients strongly influenced the balance between deterministic and stochastic processes (Figure 7C). Dispersal limitation increased with pH (*r* = 0.55, *R*^2^CV = 0.37, *p* = 0.001) but declined with higher conductivity and metals, suggesting that spatial isolation constrains communities only when chemical stress is low. Homogeneous selection was positively associated with Mn, Zn, and Cd (*R*^2^CV = 0.25–0.37) and negatively with pH (*r* = −0.31, *p* = 0.01), confirming that extreme acidity and metals act as strong environmental filters. Unexpectedly, drift followed a similar pattern, showing a strong negative correlation with pH (*r* = −0.73, *R*^2^CV = 0.57, *p* = 0.001) and positive associations with metals and ORP. This suggests that under the harshest conditions, reduced effective population sizes and intensified demographic stochasticity amplify the role of drift, leading to elevated random fluctuations despite strong selection pressures. Together, these results reveal a dynamic balance between stochastic and deterministic processes in AMD ecosystems. Drift and homogeneous selection jointly dominate under extreme stress, reflecting the combined effects of demographic instability and strong filtering by acidity and metals. In contrast, dispersal limitation becomes the primary driver under milder conditions, as spatial barriers outweigh selective constraints. This interplay highlights that community assembly in AMD systems is governed by shifting combinations of filtering, drift, and dispersal rather than by a single process.

Our findings extend previous observations from AMD lakes (She et al., 2023), groundwater habitats (Ning et al., 2024), and global AMD sites (Liu et al., 2025). We show that drift can increase under extreme stress due to reduced effective population sizes, and that the relative influence of dispersal limitation vs. selection depends strongly on the severity of acidity and metal load. These insights underscore that in extreme AMD systems, environmental filtering and stochasticity act in concert, rather than in opposition, to shape microbial communities. Because AMD geochemical variables are often highly correlated (e.g., metals and pH), we interpret these associations as reflecting suites of environmental drivers, which is ecologically realistic for such systems.

## Conclusion

This study reveals that acid mine drainage microbial communities are structured by a dynamic interplay between extreme environmental filtering and spatial processes, with pH and metal gradients as the dominant selective forces. By resolving microdiversity at the ASV level, we show that a small, persistent set of specialized acidophiles consistently thrive under harsh conditions, including both classical taxa and previously underreported uncultured lineages. Within this persistent community, fine-scale niche partitioning and contrasting patterns of phylogenetic-niche relationships across genera highlight diverse evolutionary strategies for coexistence. For example, *Acidithiobacillus* exhibits competition-driven clustering, while *Leptospirillum* shows complementarity. Our assembly mechanism analysis reveals that deterministic filtering and stochastic drift dominate under severe stress, while dispersal limitation becomes increasingly important under less extreme conditions. Together, these findings advance understanding of AMD ecology by uncovering the microdiversity and assembly principles that underpin community persistence and resilience in extreme environments.

Future research should explicitly link microdiversity to functional traits through integration of genomic and transcriptomic data that identifies the gene-level basis of niche differentiation. Comparative analyses across geographically distinct AMD systems will be essential to determine whether the identified assembly rules and persistence strategies represent general ecological principles. Such efforts will refine predictions of microbial responses to environmental change and support the targeted use of acidophilic consortia in bioremediation and bioengineering applications.

## Data Availability

The sequencing data generated in this study have been deposited in the NCBI Sequence Read Archive (SRA) under accession numbers SRR35104735-SRR35104846 (see Table S1 for the identifiers of each sample), in the BioProject PRJNA1309297.
