# Supplementary material for: Microdiversity and fine-scale niche differentiation support persistence and coexistence of acidophiles in acid mine drainage
Source: Front Microbiol. 2025 Dec 3;16:1697424. doi: 10.3389/fmicb.2025.1697424 (PMC12709672; doi:10.3389/fmicb.2025.1697424)
Supplement: Supplementary file 2 [file Data_Sheet_1.pdf]

Microdiversity and fine-scale niche differentiation support persistence and coexistence of acidophiles in acid mine drainage

Alejandro Palomo[1,2], Bowei Li[1,2], Zhixiong Huang[1,2], Yunjie Ma[1,2], Wenle Peng[1,2,3], Weishi Wang[1,2], Yi Wen[4*], Lihong Yang[1,2,3*]

[1]State Key Laboratory of Soil Pollution Control and Safety, School of Environmental Science and Engineering, Southern University of Science and Technology, Shenzhen 518055, China
[2]Guangdong Provincial Key Laboratory of Soil and Groundwater Pollution Control, School of Environmental Science and Engineering, Southern University of Science and Technology, Shenzhen 518055, China
[3]MEE Key Laboratory of Integrated Surface Water-Groundwater Pollution Control, School of Environmental Science and Engineering, Southern University of Science and Technology, Shenzhen 518055, China.
[4]Technical Centre for Soil, Agriculture and Rural Ecology and Environment, Ministry of Ecology and Environment, Beiing 100012, China

*Correspondence:
yanglh@sustech.edu.cn
wenyi@tcare-mee.cn

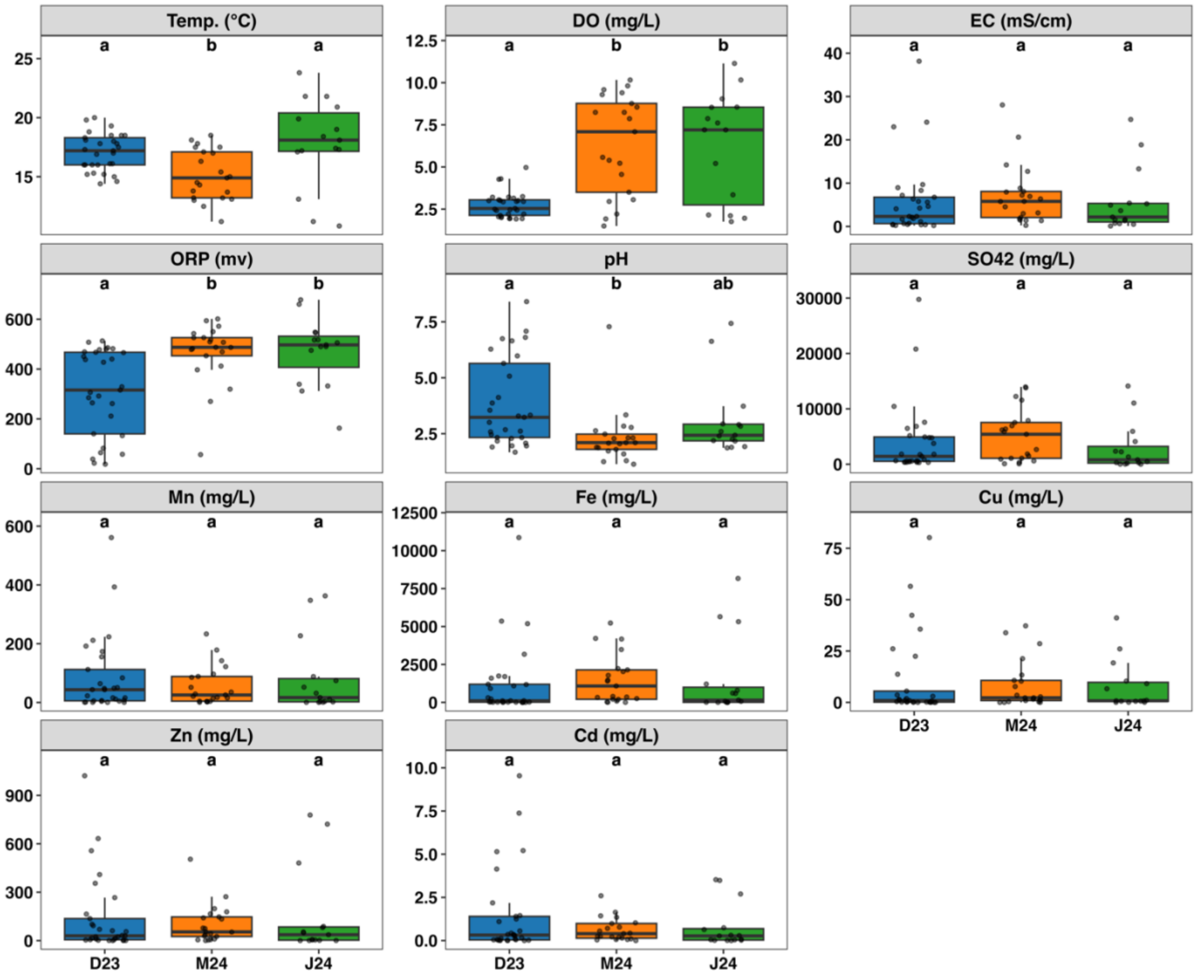

Figure S1. Physicochemical parameters of the water samples.

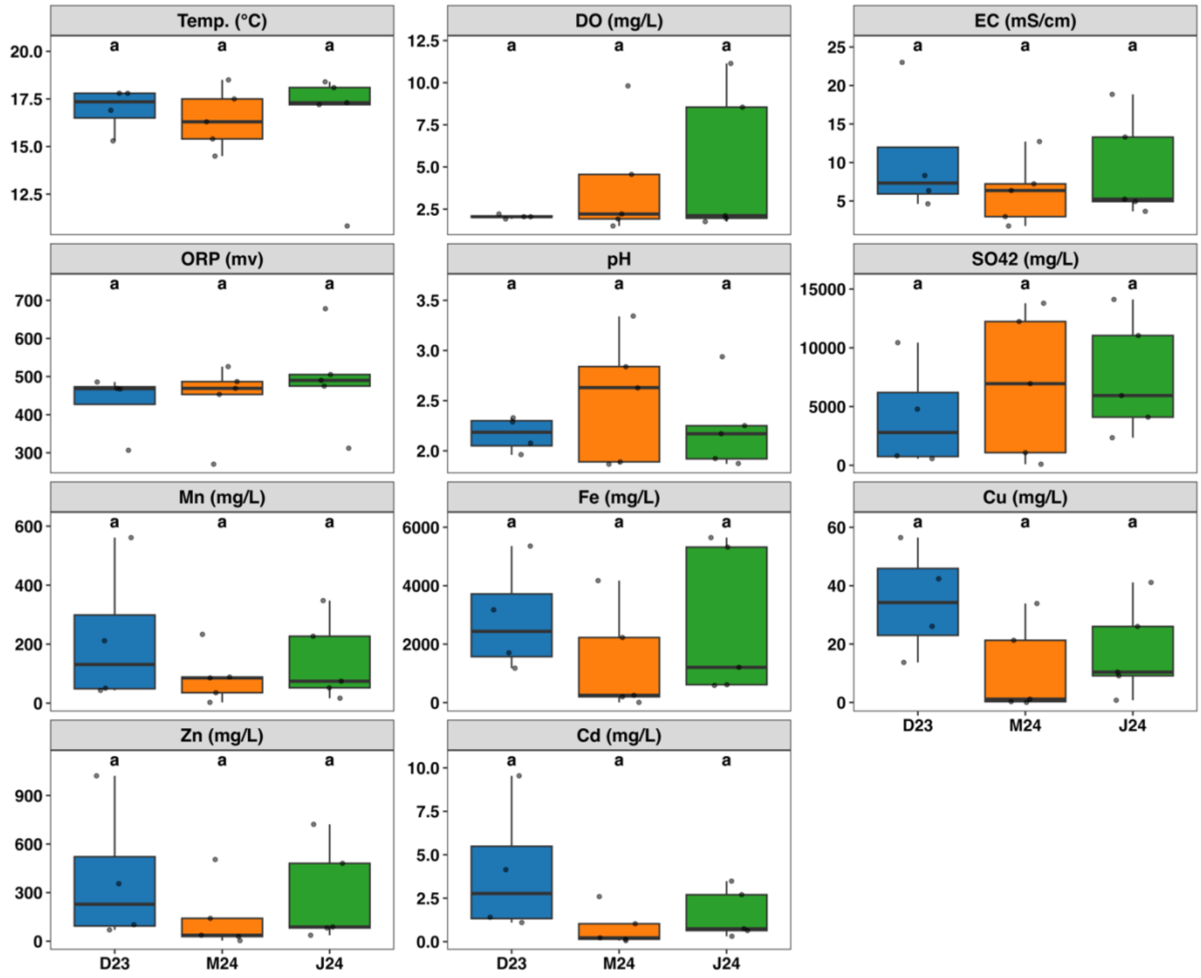

Figure S2. Physicochemical parameters of the acidic water samples (pH < 4) consistently monitored across the three campaigns (n= 5).

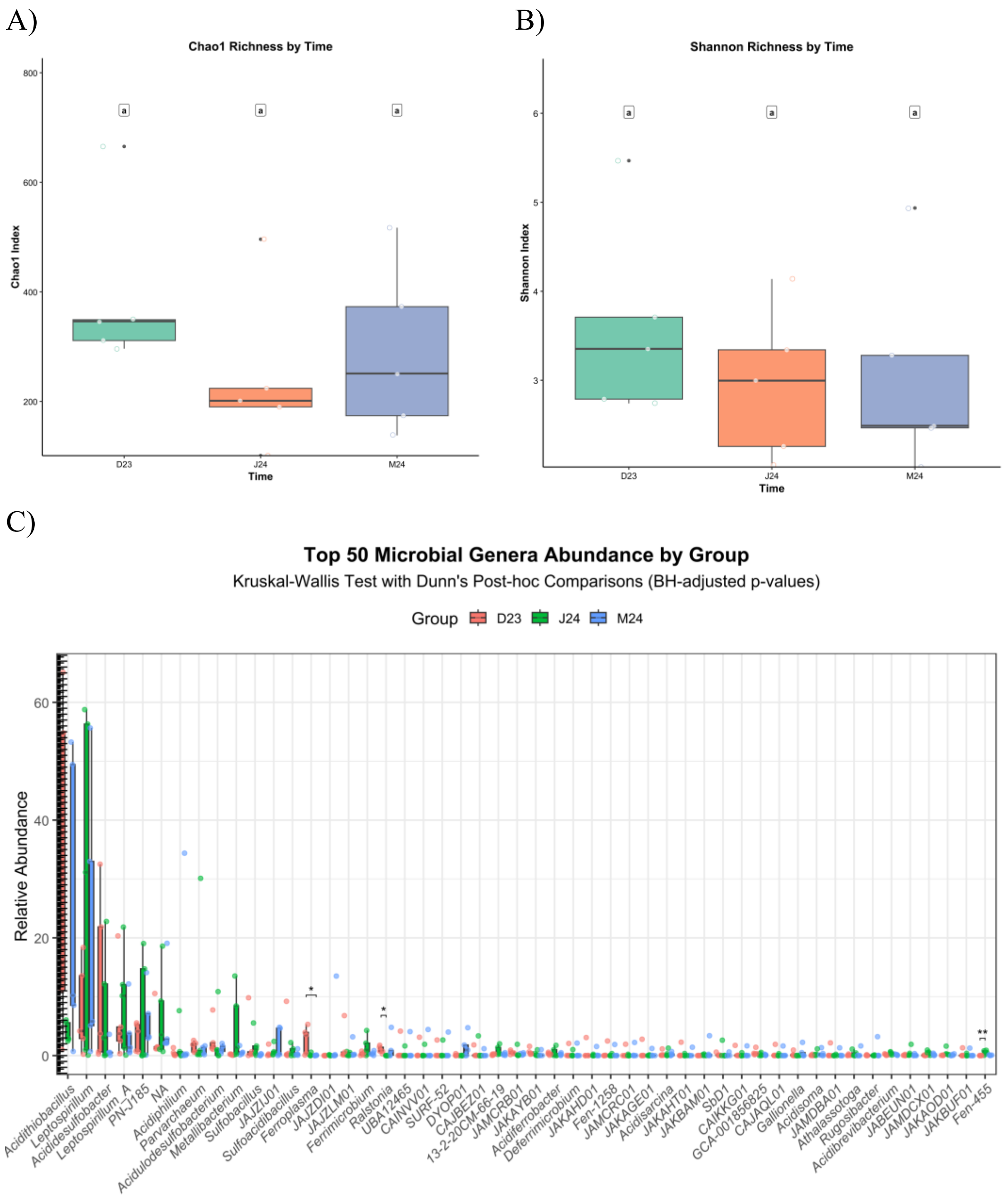

Figure S3. Microbial community richness (A), diversity (B), and composition (C) at the sampling sites consistently monitored across the three campaigns (n= 5).

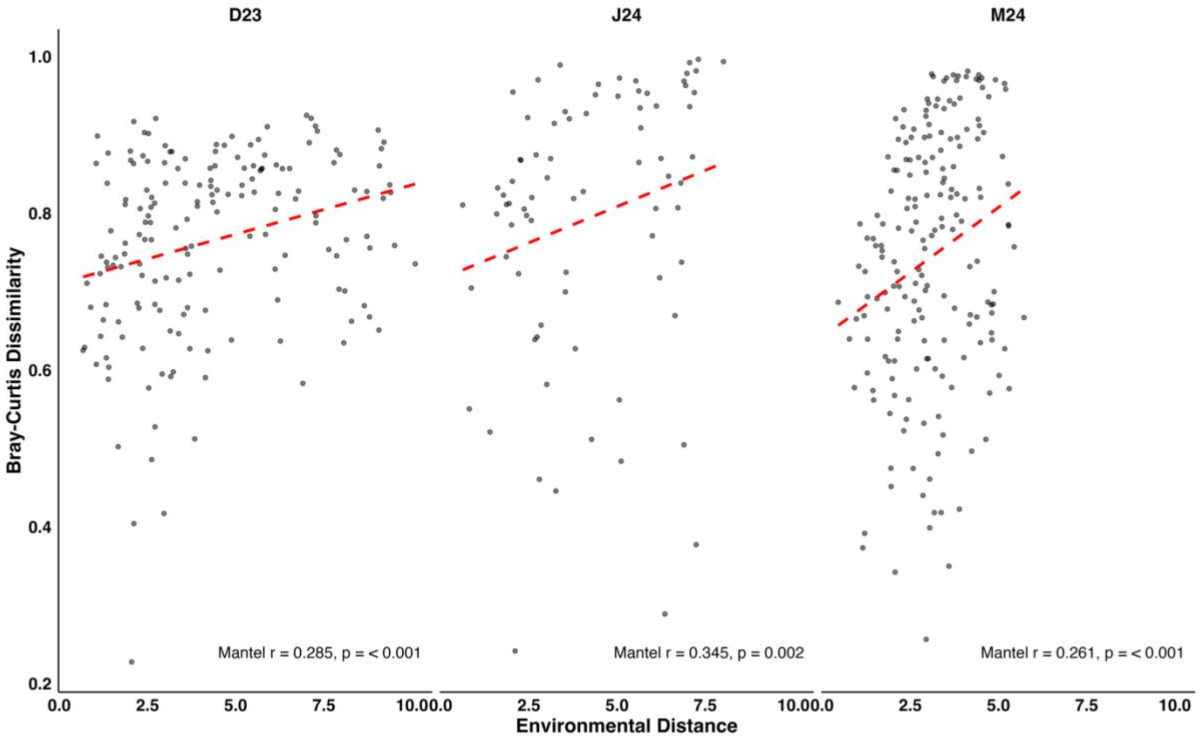

Figure S4. Relationship between community dissimilarity (measured as Bray-Curtis distances) and environmental distance (computed as Euclidean distance based on scaled values of the physicochemical parameters) in low pH water samples.

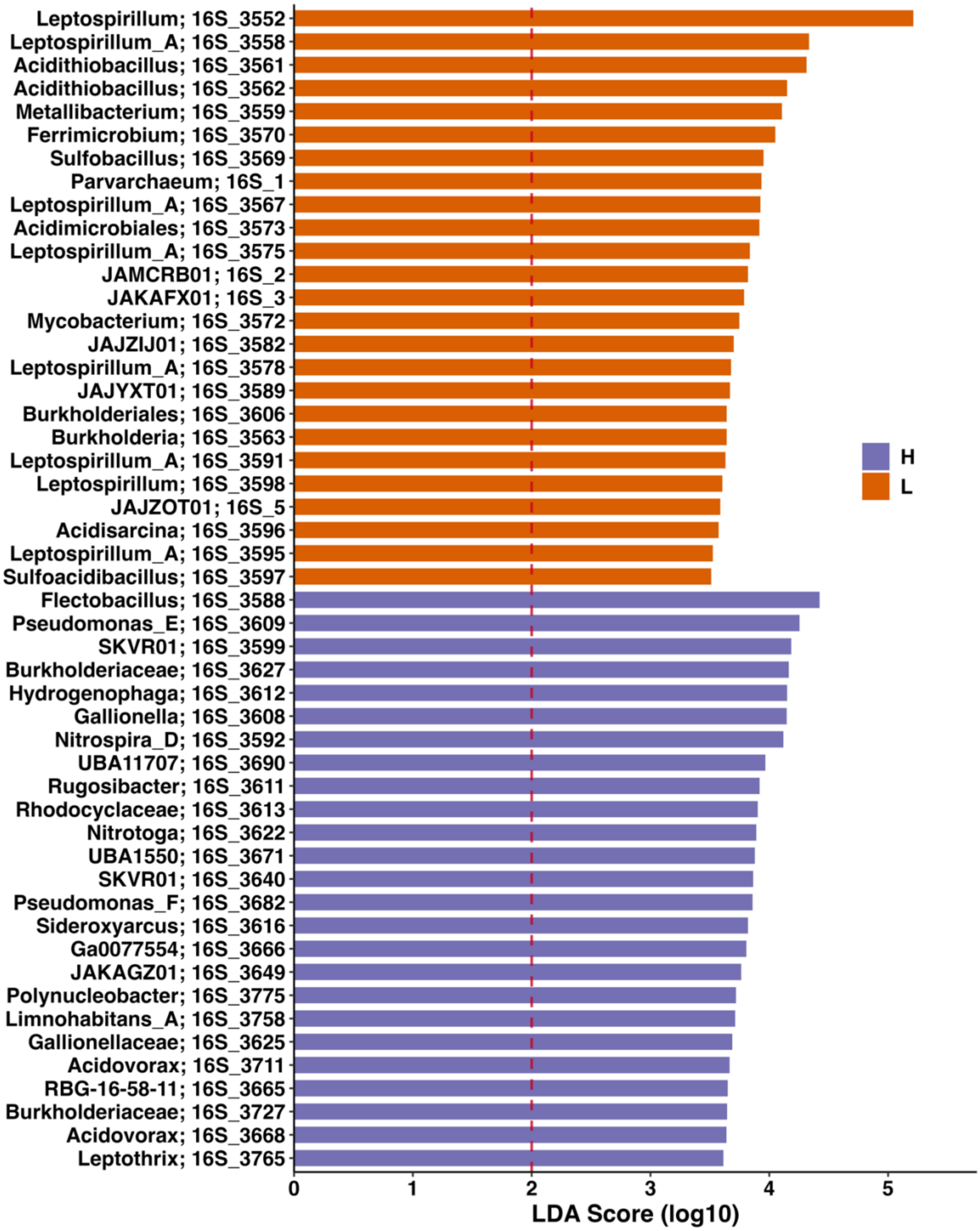

Figure S5. Linear discriminant analysis (LDA) effect size (LEfSe) showing ASVs with significant differences among samples based on pH level (L: < 4; H: > 4). LDA score is shown at logarithmic scale, and only LDA scores > 2 are considered significant. Only the top 25 more abundant ASVs in each case are presented in the plot.

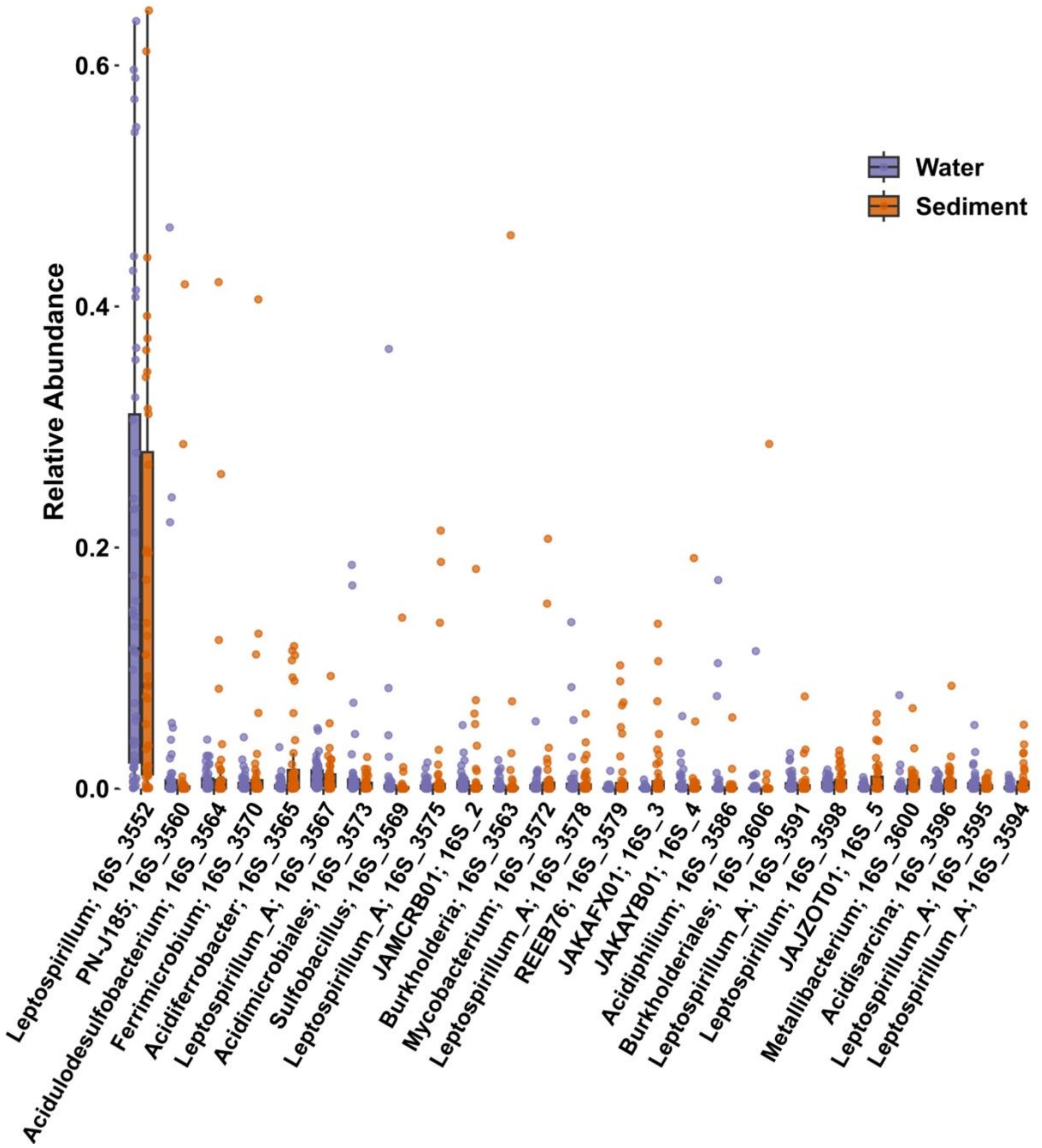

Figure S6. Relative abundance of the top 25 ASVs with non-significant differences between water and sediment samples (p.adj > 0.05). Significant differences were assessed with Wilcoxon rank-sum tests with Benjamini-Hochberg FDR correction.

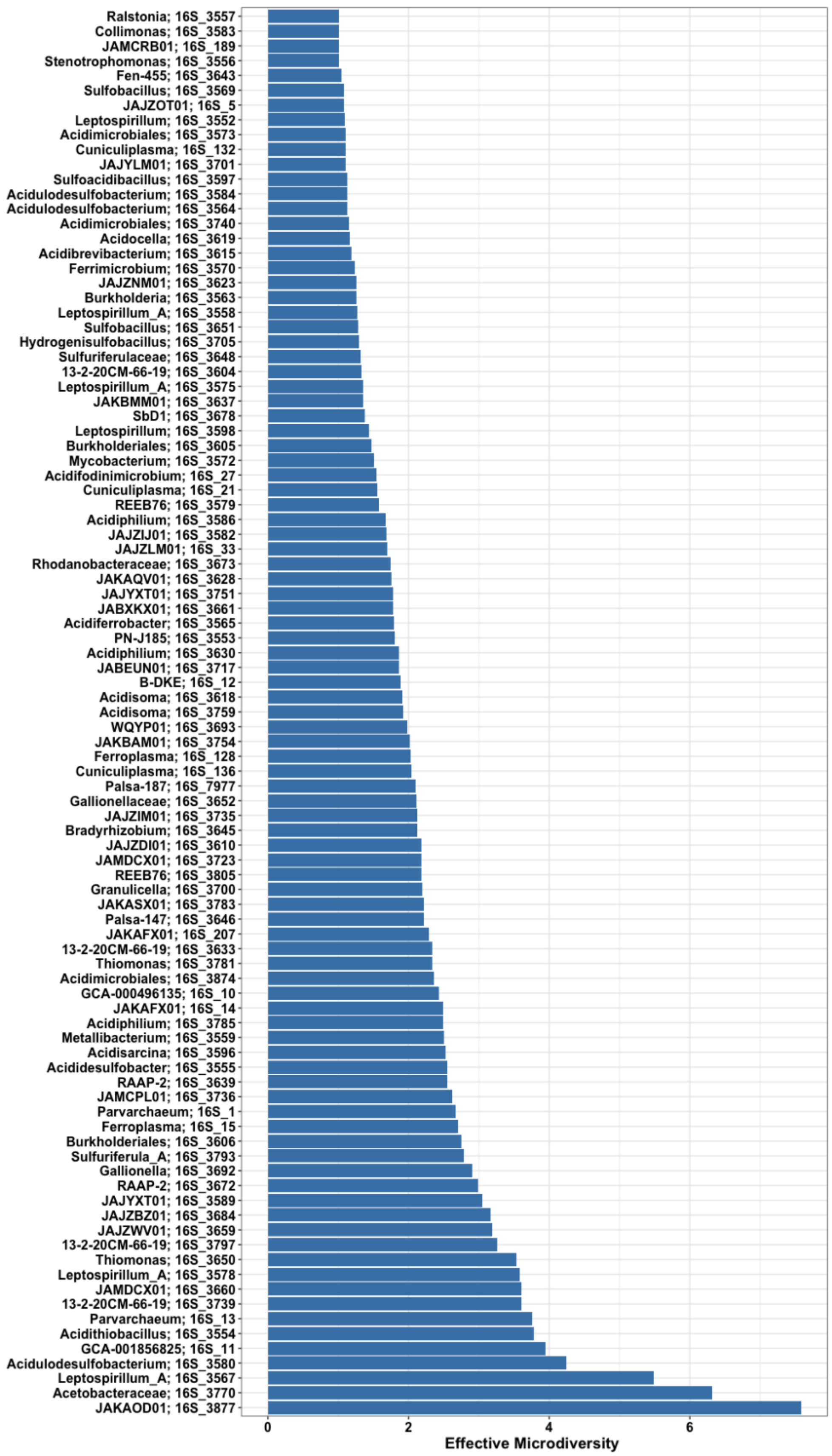

Figure S7. Effective microdiversity of 97 OTUs with total reads greater than 5000 across the low pH water samples.

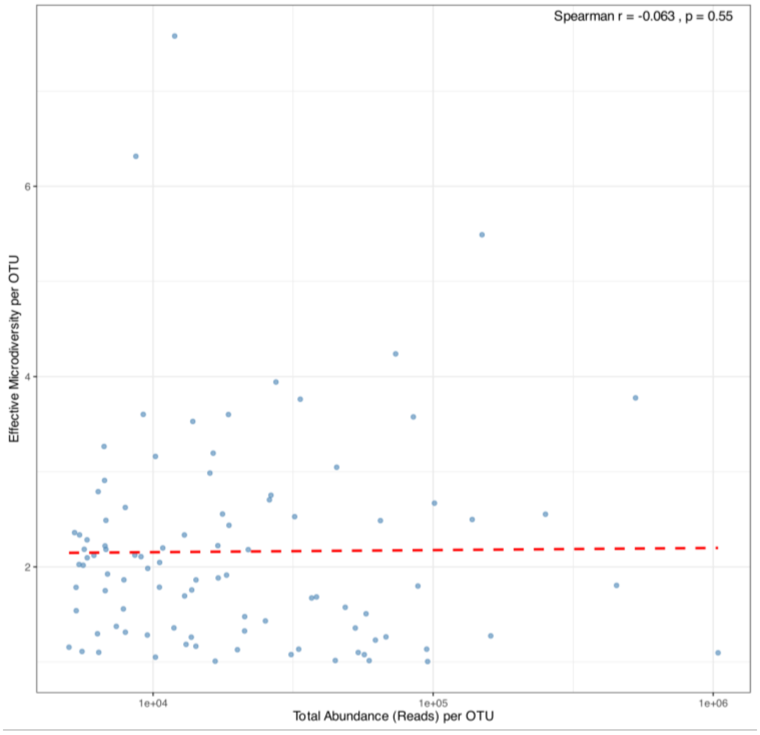

Figure S8. Relationship between the Effective microdiversity and the total abundance of 97 OTUs with total reads greater than 5000 across the low pH water samples.

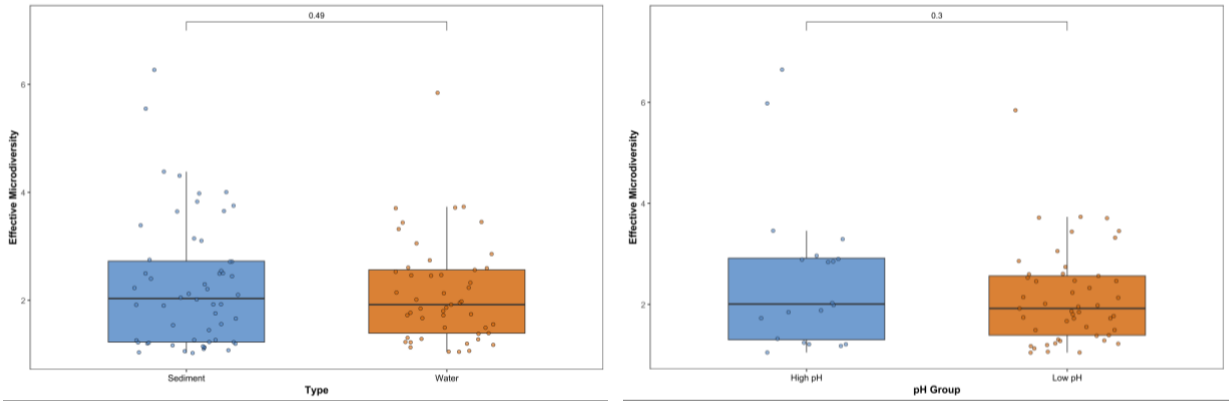

Figure S9. Effective microdiversity of OTUs calculated across: left) sediment and water samples; right) low (< 4) and higher (> 4) pH.

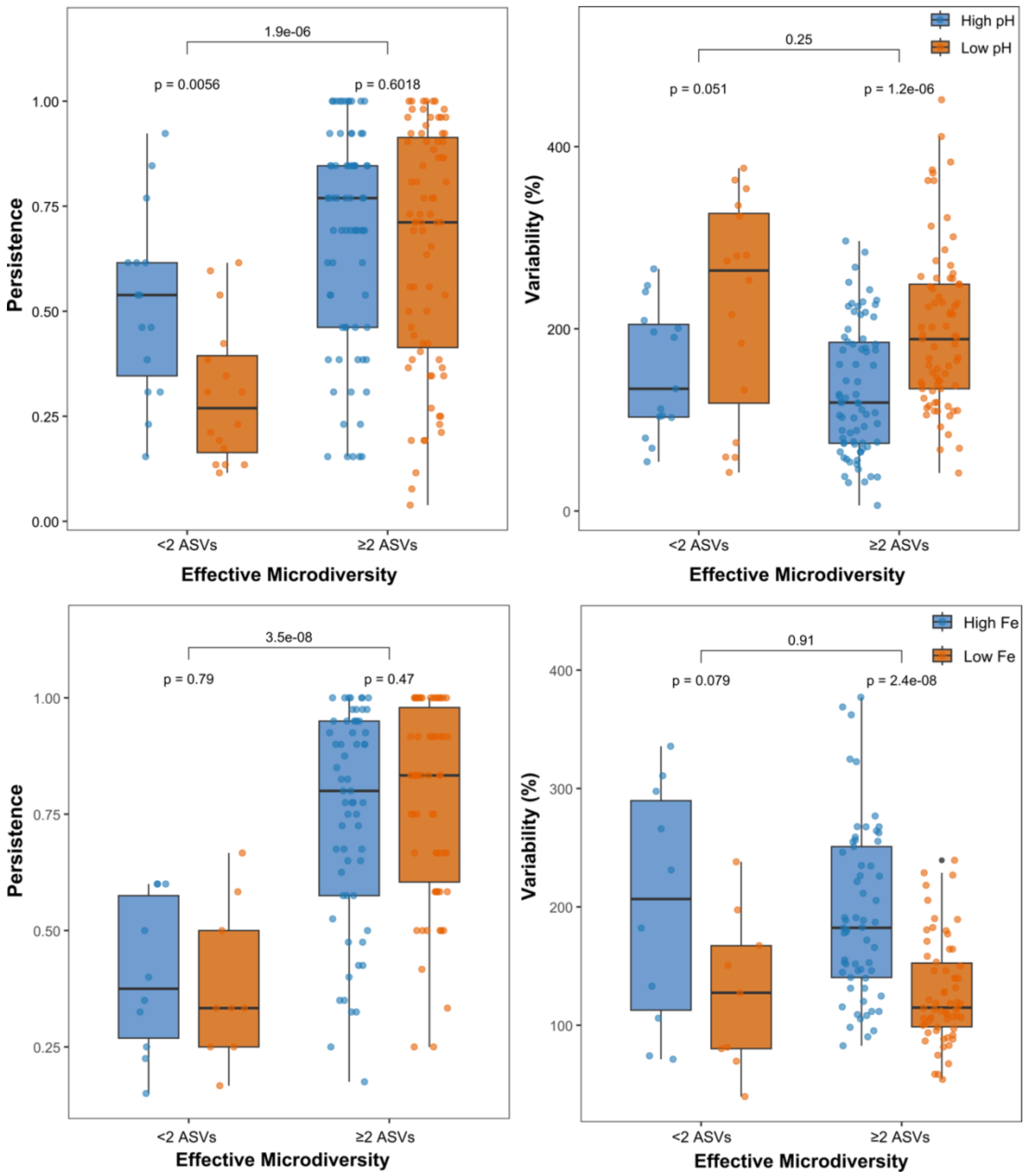

Figure S10. Effect of microdiversity on OTU persistence and variability across samples cluster based on pH levels (top; High (>4) and Low (< 4)) or Fe loads (bottom; High (> 100mg/L) and Low (< 100mg/L)).

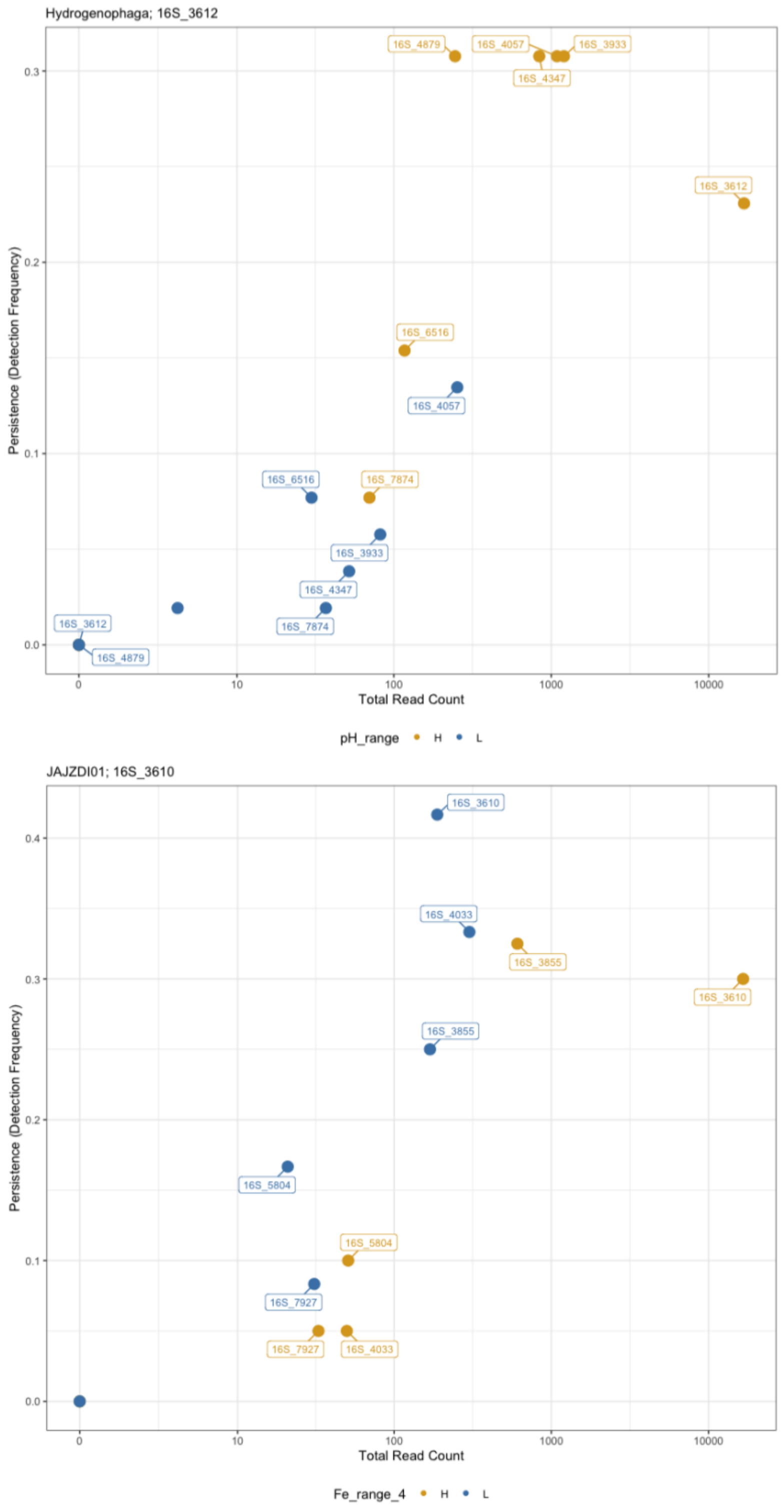

Figure S11. Scatter plots of log-transformed overall relative abundance (read counts) and persistence for ASVs within OTU in: top) low (blue) and high (yellow) pH water samples; bottom) low (blue) and high (yellow) Fe-containing water samples. Each dot represents a single ASV, and the most abundant ASVs are labeled with the ASV-IDs.

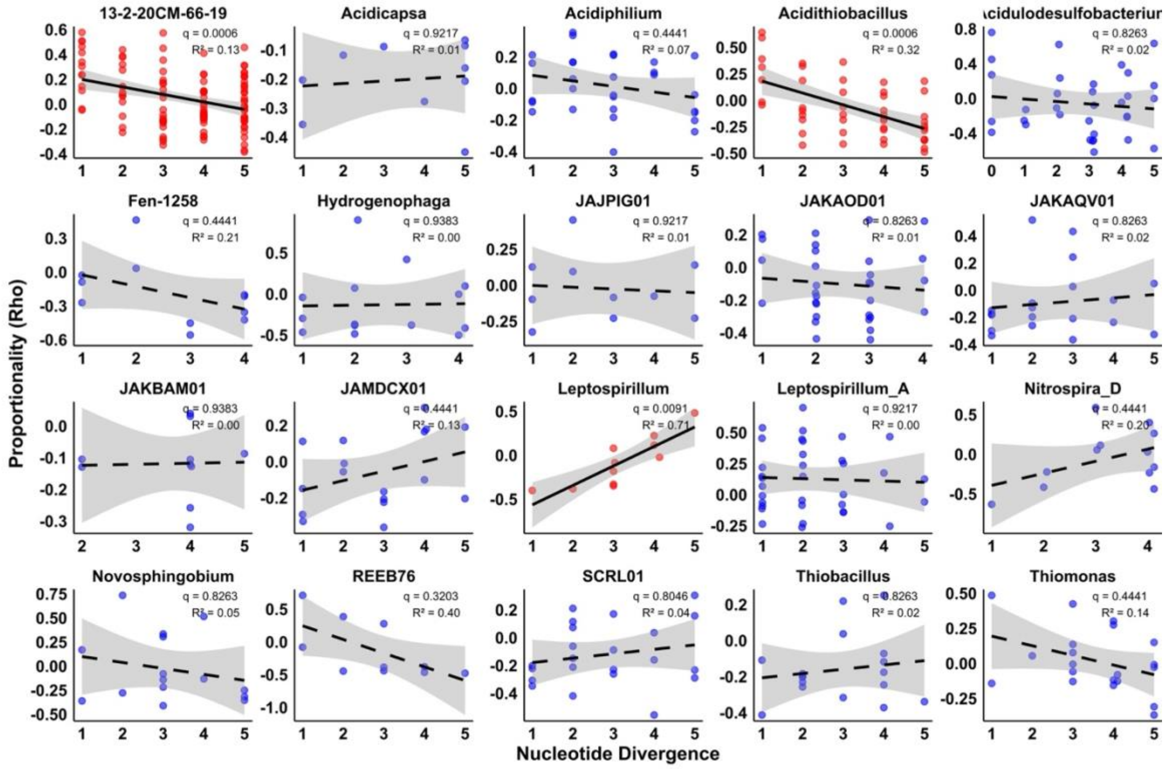

Figure S12. Relationship between nucleotide divergence and niche similarity (Rho) within genera. Red points indicate statistically significant relationships (FDR-adjusted $p < 0.05$).

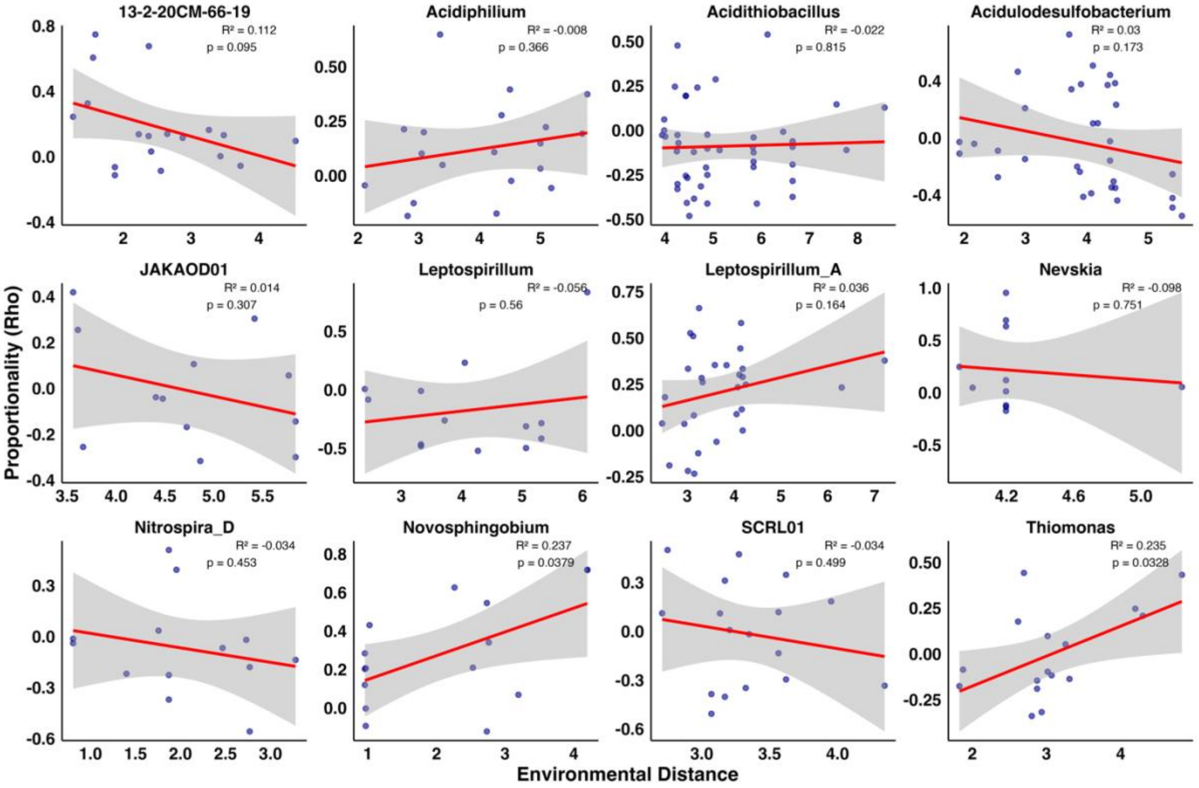

Figure S13. Relationship between environmental distance and niche preference (evaluated as proportionality (Rho)) in the low pH water samples. Only genera with at least 10 ASV pairs at less than 5 nucleotide divergences were evaluated. Gray and black lines represent the linear relationship between the two variables (black indicates statistical significance).

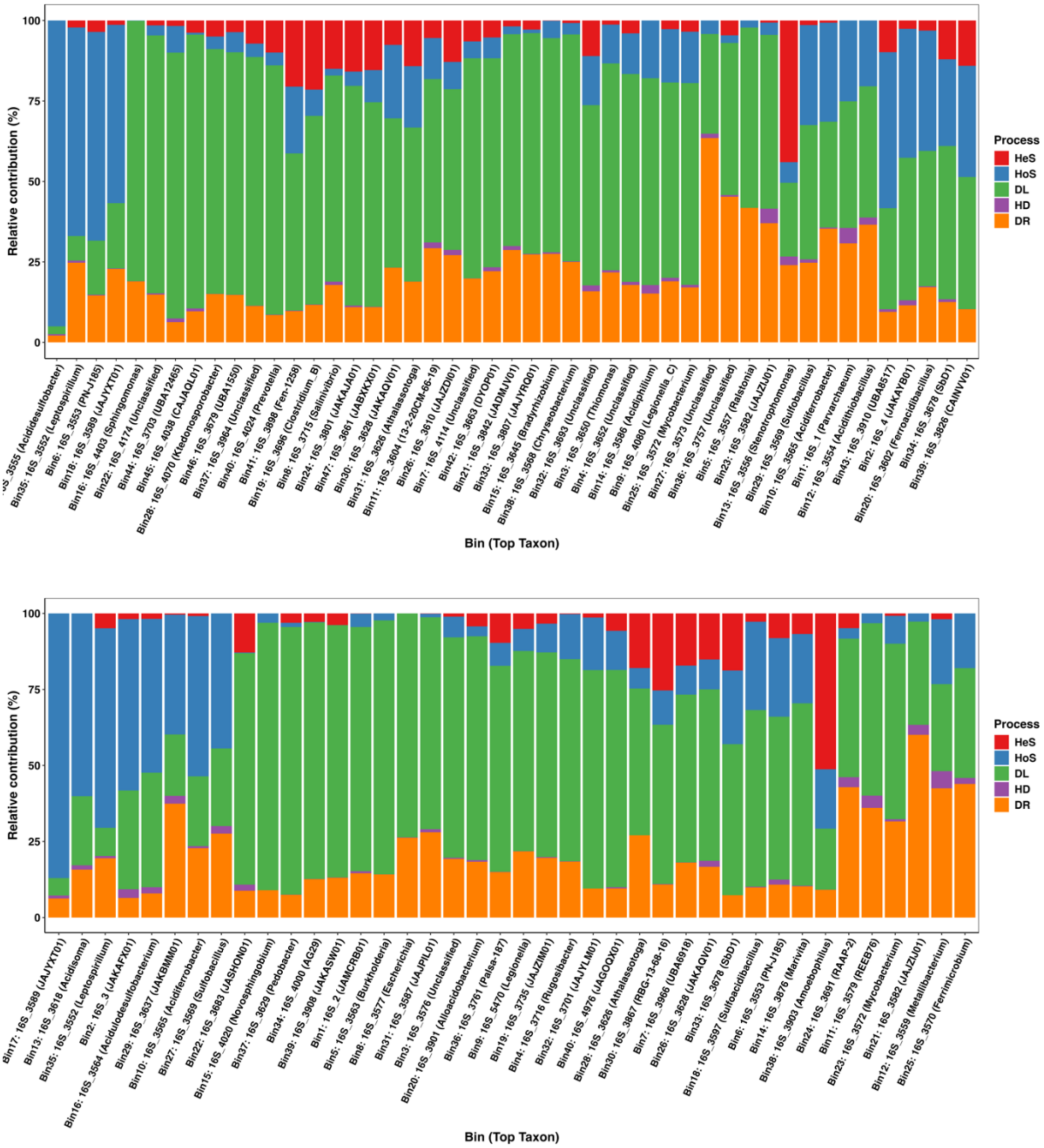

Figure S14. Relative importance of different processes in each phylogenetic bin from iCAMP analysis in water (top) and sediment. (bottom) samples.
